# Choice of primary healthcare providers among population in urban areas of low- and middle-income countries: a systematic review of literature

**DOI:** 10.1186/s12875-026-03261-1

**Published:** 2026-03-18

**Authors:** Md. Zahid Hasan, Khadija Islam Tisha, Md Golam Rabbani, Natalie King, Edward JD Webb, Zahidul Quayyum, Tim Ensor

**Affiliations:** 1 Nuffield Centre for International Health and Development, Leeds Institute of Health Sciences, University of Leeds, Leeds, LS2 9NL UK; 2 Academic Unit of Health Economics, Leeds Institute of Health Sciences, University of Leeds, Leeds, LS2 9NL UK; 3 Health Economics and Financing, Health Systems and Population Studies Division, icddr,b, Dhaka, 1212 Bangladesh; 4 BRAC James P Grant School of Public Health, BRAC University, Dhaka, Bangladesh

**Keywords:** Primary healthcare, Discrete choice experiments, Revealed preferences, Stated preferences, Urban health

## Abstract

**Background:**

Strengthening urban primary healthcare (PHC) systems in low- and middle-income countries (LMICs) is essential to effectively serve the growing urban poor. Such reforms should incorporate patient opinions to ensure accessible and affordable services. However, there is limited evidence on patient preferences for PHC providers in these settings. We aimed to conduct a systematic review of the evidence on the attributes reported by patients when choosing PHC providers in urban LMICs.

**Methods:**

A search was performed across MEDLINE, Embase, Global Health, Web of Science, PsycINFO, and Scopus bibliographic databases, as well as grey literature sources, from their earliest entries until March 30, 2024. Studies examining the revealed or stated preferences of populations for PHC providers in urban LMIC settings were included. We descriptively analysed and compared these studies, assessing their methodological quality using checklists for Conjoint Analysis studies and the Mixed Methods Appraisal Tool.

**Results:**

Our findings are reported according to PRISMA guidelines. The search yielded 5,089 citations, of which 28 met the eligibility criteria for this review. We identified 203 attributes across selected studies. The most frequently reported attributes were cost of services (*n* = 25) and distance/proximity (*n* = 24), followed by provider behaviour/attitude (*n* = 19) and quality of care (*n* = 17). Most studies identified the availability of medicine/equipment, distance/proximity, healthcare provider type, and quality of care as the most valued attributes. Eighteen studies examined preference heterogeneity, considering socioeconomic factors such as education, gender, age, severity of illness, and income, with severity of illness being the most significant factor.

**Conclusions:**

Prioritising patient preferences in health system reforms is essential for equitable and sustainable healthcare in rapidly urbanising LMICs. Our review highlights the most valued attributes in preference studies, which will help policymakers and researchers tailor better PHC interventions to meet urban community needs and guide future studies on PHC preferences in similar settings.

**Supplementary Information:**

The online version contains supplementary material available at 10.1186/s12875-026-03261-1.

## Introduction

Primary healthcare (PHC) is considered the best platform for providing basic healthcare services to the population and performing essential public health functions [1]. It is one of the key elements of a country’s health system and provides various types of services, including health promotion, disease prevention, treatment, rehabilitation, palliative care, and more. PHC also ensures that healthcare is delivered in a way that is centred on people’s needs and respects their preferences [2]. It serves as a cornerstone of an effective healthcare system that helps improve population health cost-effectively and equitably [3]. In the 1978 Alma-Ata Declaration, PHC was set as a global priority to protect and promote health for all people worldwide [4]. PHC also plays a central role in achieving Universal Health Coverage (UHC), aiming to ensure that all people have access to the full range of quality health services they need, when and where they need them, without financial hardship [5]. More recently, the 2018 Astana Declaration on PHC made a similar call for universal coverage of basic healthcare for the population throughout their life, emphasising essential public health functions, community engagement, and a multisectoral approach to health [6]. Rapid and uncontrolled urbanisation imposes challenges to urban PHC systems in many low- and middle-income countries (LMICs) to meet the increased healthcare demand of the urban population, especially for the poor [7]. Urbanisation has also created significant barriers to achieving UHC, as health systems often struggle to expand equitable PHC coverage in rapidly growing urban settings, and a challenge further compounded by lack of public-private collaboration [8, 9].

Among the existing qualified urban PHC practitioners, a high percentage are likely to be engaged in private practice, limiting accessibility to poor people. The situation is worse in countries where the urban PHC system is poorly structured, resulting in fewer public providers in urban compared to rural areas [10, 11]. Furthermore, the demographic transition and the rising prevalence of non-communicable diseases have amplified the demand for healthcare services in both rural and urban areas [12]. A considerable proportion of urban people live in slum areas, which often lack the most basic human needs such as clean water, sanitation, and adequate housing. These populations are more vulnerable to illness and frequently experience worse health outcomes than their rural counterparts [13].

In many LMICs, PHC has been identified as a major priority by health system planners. Efforts are focused on reorienting existing PHC systems to achieve UHC by prioritising the delivery of efficient PHC, strengthening effective and patient-centred care, and reducing inequalities in healthcare access [14]. In response to the greater need for PHC services, especially among urban communities, reforming the urban PHC system is essential for delivering need-based healthcare services efficiently [15]. Such reforms should prioritise incorporating patients’ opinions in the redesign of services to ensure that service delivery effectively meets the needs of the urban population in a timely and cost-efficient way. In this context, policymakers need to understand patients’ choices and preferences regarding various aspects of PHC services for redesigning and delivering these services for the urban poor communities. A systematic review of the preferences, either stated preferences (SP) or revealed preferences (RP), of the urban poor population for PHC providers in urban areas, can help identify the key drivers of provider selection and design more responsive health service delivery models.

To date, two systematic reviews on patient preferences for PHC services have been conducted [16, 17], focusing on SP only. Kleij et al. 2017 synthesised attributes from 18 studies published between 2006 and 2015, but did not examine preference heterogeneity. Lim et al. 2022 expanded the earlier review and synthesised attributes from 35 studies published before December 2021 and examined preference heterogeneity. However, three important gaps remain in the existing evidence: (1) neither review included RP studies nor compared attributes derived from RP and SP studies; (2) neither specifically focused on PHC preference in urban LMIC contexts, where urban-rural differences in socioeconomic factors shape distinctive choices [18], and (3) evidence on PHC preferences specific to urban populations in LMICs has not been systematically synthesised. This systematic review addresses these gaps by synthesising evidence specifically on PHC provider preferences in urban LMIC settings in three ways. First, we focus exclusively on urban areas in LMICs, which present distinctive healthcare choice environments compared to rural settings, such as diverse provider markets, population density, informal sector presence, and out-of-pocket (OOP) payment reliance. Second, we include both SP and RP studies, enabling comparison between what people state they prefer in hypothetical scenarios versus the attributes that influence their actual healthcare-seeking behaviour. Third, we examine preference heterogeneity across socioeconomic and demographic factors to understand how preferences vary within urban populations. A synthesis of evidence for PHC attributes for the urban health system will help future research and policy decisions for effectively designing and delivering healthcare services to the urban population.

## Objective

We aimed to synthesise existing evidence on patients’ preferences for PHC providers in urban areas of LMICs to identify lists of attributes specific to the urban population.

### Research questions

This systematic review focused on the studies that elicit patients’ or the population’s preferences, RP or SP, for urban PHC in LMIC settings. The specific research questions are:

1. What are the attributes/characteristics of urban PHC providers that influence whether the population uses their services?
2. What are the attributes/characteristics of PHC providers that are identified as most important to the population?

## Methods

The systematic review was prospectively registered in the International Prospective Register of Systematic Reviews (PROSPERO) database (CRD42023409720) (Date of registration: 13 April 2023) and is reported in accordance with the Preferred Reporting Items in Systematic Reviews and Meta-Analyses (PRISMA) guidelines [19] (Appendix 1).

### Search strategy

We searched selected databases, as well as grey literature sources, from their earliest entries until March 30, 2024. We developed a search strategy for identifying the relevant literature on the preference of PHC providers using a combination of Medical Subject Headings (MeSH), keywords, and text words. The search terms were adapted from previously published systematic reviews on preference for PHC [16, 17] as well as reviews on PHC in LMICs [20, 21], and preference studies [22]. The scope of this systematic review was finalised in consultation with Author7, Author6, and Author5. An information specialist (Author 4) guided the finalisation of the search strategy. Initially, the search strategy was developed for Medline and translated into other relevant electronic databases. These included EMBASE, Global Health, Web of Science, PsycINFO, and Scopus. We explored relevant studies and reports from ProQuest Dissertations and Theses, Google Scholar, Social Science Research Network (SSRN), Global Index Medicus, 3ie, and World Bank. Additionally, we manually reviewed the bibliographies of included studies to identify relevant articles that met the inclusion criteria (a detailed presentation of the search strategy is included in Appendix 2). However, we did not conduct systematic backward and forward citation tracking of the included articles.

### Inclusion and exclusion criteria

The review included studies that examined the revealed or stated choices or preferences of populations for PHC providers in urban LMIC settings. For this review, PHC was defined as first-contact or general care delivered at the primary level healthcare facility, including services provided through community-based facilities, primary care clinics, and outpatient departments. Studies focusing exclusively on secondary, tertiary-level, or specialised care were excluded. Urban settings were defined based on the classification or reporting used by the primary study authors. Countries were classified as LMICs according to The World Bank income classification applicable at the time the review was conducted. We included a range of study types, including SPs, quantitative cross-sectional studies, and qualitative studies. Peer-reviewed articles, grey literature, published reports, and conference abstracts were considered if published in English, while study protocols, systematic reviews, newspaper articles, letters, editorials, personal communications, and commentaries were excluded. The age of study participants was restricted to 18 years and above in the included studies. Studies that considered preferences from both individual and societal perspectives were included. Additionally, studies reporting outcomes related to preference attributes, attribute levels, and the development of preference sets on PHC were included. Studies were excluded if they considered shared decision-making (including providers in the decision-making process) options in the preference. Furthermore, studies focusing solely on rural populations or any preference studies that did not focus on PHC services were excluded to align with review objectives. A detailed list of inclusion and exclusion criteria is attached as Appendix 3.

### Study selection

We applied a three-stage screening process to select studies for review and data extraction. Initially, studies were chosen based on predefined eligibility criteria to ensure consistency among reviewers. Subsequently, two independent reviewers (Author1 and Author2) screened the titles and abstracts obtained from the search to identify potentially relevant studies. Full-text articles or documents of the identified studies were then retrieved and reviewed. Any discrepancies between reviewers were resolved through discussion and consensus; wherever disagreements persisted, a third reviewer (Author3) was consulted. All the selected studies were discussed and finalised for full-text review in consultation with Author7, Author6, and Author5. The selection process was documented and reported using a PRISMA flow diagram. Screening and selection of relevant studies were managed using Covidence [23].

### Data extraction

We developed a data extraction template using Microsoft Excel. This template underwent piloting by two reviewers (Author1 and Author2) and subsequent review and finalisation by another reviewer (Author3). From eligible studies, data were extracted on common information such as study population, study settings (e.g., rural–urban or urban), country where the study was conducted, types of studies (e.g., DCE, quantitative, qualitative, mixed-methods), type of healthcare visit (e.g., inpatient or outpatient), context of the health system, methods of data collection, authors, and year of publication. For quantitative and qualitative component of mixed methods studies, data extraction included different attributes/ characteristics (e.g., distance, waiting time) related to the PHC provider preference, levels of the examined attributes, which attributes/ characteristics were reported as most important attributes/ characteristics, and heterogeneous factors affecting the preferences of the population. Additionally, for quantitative DCE studies, we extracted the methods used to identify attributes and their corresponding levels, to generate choice sets, and the types of analyses (e.g., the statistical model used) reported. For the qualitative component of mixed-methods studies, themes or subthemes relevant to the review questions were extracted and supported with illustrations (i.e., a direct quotation from a participant, an observation, or other supporting data from the reviewed studies) to preserve the context of the findings.

### Quality assessment

To critically appraise the validity and identify potential sources of bias in the included studies, we used the ISPOR (International Society for Pharmacoeconomics and Outcomes Research) checklist and the MMAT (Mixed Methods Appraisal Tool). We selected the ISPOR checklist for SP studies as it is the internationally recognised standard specifically designed for evaluating stated preference studies, such as discrete choice experiment and conjoint analysis [24]. For RP studies, we selected the MMAT because it provides a consistent appraisal framework for the diverse study designs in our review (quantitative cross-sectional, qualitative, and mixed-methods) [25]. Prior to assessment, reviewers were familiarised and calibrated on these tools to ensure consistent application. The quality of SP studies was assessed using the ISPOR checklist [24], comprising ten items, each with three criteria. Independent reviewers evaluated each criterion as “Yes,” “Partial,” or “No.” Following this assessment, each item was rated accordingly. For summarising quality score, only criteria rated as “Yes” were counted, and the total number of “Yes” ratings was divided by the total number of ISPOR criteria assessed for each study and expressed as a percentage.

As for studies categorised as RP, the latest version of the MMAT (version 2018) was employed [25]. This tool is designed to assess the quality of quantitative, qualitative, and mixed-method studies, featuring two screening questions and five criteria for each study type scored as ‘yes,’ ‘no,’ or ‘cannot tell.’ All included RP studies underwent appraisal based on two initial screening questions: a) whether the study had clear research questions, and b) whether the collected data allowed addressing their respective research questions to determine the feasibility or appropriateness of further methodological quality appraisal. Studies with responses of ‘no’ or ‘cannot tell’ to both of the above two questions were excluded from further evaluation. The total percentage quality score for each study was calculated based on the MMAT scoring guide, with only the number of items scored ‘yes’ contributing to the overall score [26]. For this review, scores of ≤ 60% were deemed ‘low quality,’ while scores falling within the range of 61–80% were considered ‘average quality.’ Scores ranging from 81% to 100% were classified as ‘high quality’ for both SP and RP studies. Given the subjective nature of critical appraisal, the quality assessment of included studies was independently conducted by Author1 and Author2, with any disagreements resolved through consensus or consultation with Author3. Quality assessment was conducted to transparently report the methodological rigor of included studies and allow readers to interpret findings in light of methodological limitations. All studies meeting the inclusion criteria were included in the synthesis regardless of quality scores, as our objective was to comprehensively identify preference attributes across the urban LMIC.

### Data analysis

We utilised a segregated approach to synthesise the quantitative and qualitative evidence independently before integrating their findings. The integration of attributes identified from qualitative findings with the quantitative attributes followed a data-based convergent synthesis design. Our narrative approach concentrated on the preference attributes of healthcare seeking, including factors such as distance, travel time, and costs. To offer a comprehensive overview, we tabulated the study characteristics (type of study, recruitment setting, survey administration, etc.) using numbers and percentages. Guided by Donabedian’s Structure-Process-Outcome (SPO) framework for the quality of healthcare model, the attributes were categorised into three levels: structure, process, or outcome [27]. This established framework was useful in categorising the diverse attributes identified across studies and summarising what dimensions of healthcare were important for the respondents when choosing PHC providers.

An initial categorisation of the attributes was done by Author1 and then reviewed and revised in consultation with Author7, Author6, and Author5. In cases where attributes were multidimensional and could plausibly fall into more than one category (e.g., waiting time or perceived quality of care), classification was guided by the operational definitions of the levels, as well as discussion among team members. After two rounds of discussion, the categories were revised and finalised following the consensus among the authors.

‘Structure’ encompasses factors essential to healthcare delivery, including material resources (e.g., care setting, equipment, medicine, cost), personal resources (e.g., provider availability), and organisational structure (e.g., distance, waiting time, convenience of appointment/referral). ‘Process’ denotes the activities occurring during the provision and receipt of care, such as the attitude of the care provider and the maintenance of confidentiality and privacy. ‘Outcome’ signifies the effects of healthcare delivery, encompassing optimal treatment and the likelihood of a cure. This also included the reputation of the provider, recommendations from acquaintances, the quality of care, the provider’s skill, and trust in the provider, serving as proxies for the effects of healthcare delivery.

We identified the most valued attributes based on the highest value of the coefficient reported in the included SP studies. In the case of included RP studies, the most important attributes were identified based on the highest score/ proportion /coefficients reported. Similarly, we identified the most influential socioeconomic factors/ heterogeneity of PHC provider preference based on the analysis reported in the included studies. We also included the direction of the common heterogeneous factor if reported in the included articles.

## Findings

### Study selection

**Fig. 1:**
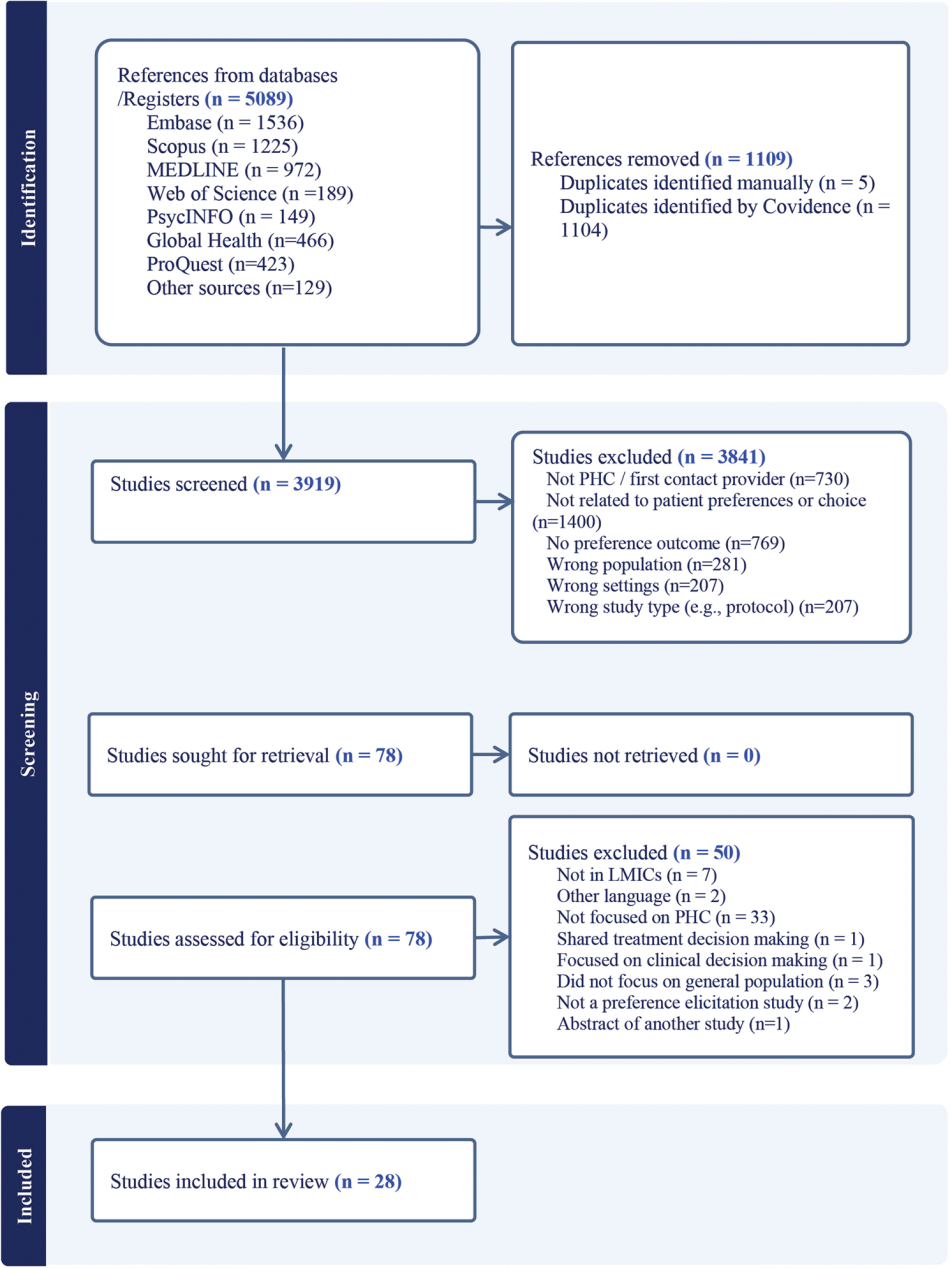
PRISMA flow diagram

The search strategy identified 5089 titles (Fig. 1). After removing duplicates, 3841 records were screened, and 28 publications met the inclusion criteria for extraction and analysis. Among the included 28 studies, half were conducted in China [28–42]. Other studies were conducted in India [43, 44], Nigeria [45, 46], Malaysia [47], Vietnam [48], Iran [49], Egypt [50], Ghana [51], Malawi [52], Sierra Leone [53], and South Africa [54, 55].

### Study characteristics

Table 1 summarises the characteristics of the included 28 studies, with detailed information on each study provided in Appendix 4. Among the 28 studies, 15 were SPs, and 13 were RPs, of which 10 were quantitative, and only three studies were mixed method designs. Study samples were primarily recruited from communities (19, including one mixed method) or healthcare facilities (8, including 2 mixed methods), and one study drew from both settings. Nearly all (24, including 3 mixed-method) studies used interviewer administration for data collection. About half of the quantitative studies (48%) and all three mixed method studies examined PHC for general conditions, chronic diseases (36%), and pediatric conditions (8%).

A diverse range of approaches was used to identify study attributes and levels, with 48% of the quantitative studies and 2 mixed-method studies employing a combination of literature reviews and qualitative interviews. Notably, nine of the quantitative studies (36%) did not explicitly report their attribute identification methods, and these were RP studies.

Out of three mixed-method studies, one study used a literature review to identify attributes only. All the studies reported a total of 203 attributes, of which 151 were in quantitative studies, and 52 were in mixed-method studies. Attributes were classified as structure (69.5% vs. 46.2%), followed by outcome (16.6% vs. 25.0%) and process (13.9% vs. 29%) attributes. Among the SP studies, eleven utilised regression models, while four employed latent class analysis. Of the 13 RP studies, 4 studies conducted descriptive analysis (2 quantitative and 2 mixed-method). Most of the extracted studies (17 out of 28) were conducted between 2019 and 2024, while 11 studies were conducted between 2007 and 2018.

**Table 1:** Study characteristics

Variables	Number of the studies (%)
Type of study	
Stated preference	15 (53.57)
Quantitative	10 (35.71)
Mixed method	3 (10.71)
Recruitment setting	
Both community and Healthcare facility	1 (3.57)
Community	19 (67.86)
Healthcare facility	8 (28.57)
Survey administration	
Interviewer administered	25 (89.29)
Self-completed	2 (7.14)
Self-completed & Interviewer administered	1 (3.57)
Disease condition	
General conditions	15 (53.57)
Chronic disease/HTN/Diabetes	9 (32.14)
Pediatric condition	2 (7.14)
Others	2 (7.14)
Methods to identify attributes & levels	
Literature review & qualitative interviews (IDI/KII/FGD)	14 (50)
Lit review & Expert opinion	2 (7.14)
Lit review, policy & FGDs/IDIs	2 (7.14)
Literature review	1 (3.57)
Not reported	9 (32.14)
Number of attributes	
up to 3	2 (7.14)
4 to 6	14 (50)
7 to 9	7 (25)
10 or more	5 (17.86)
Types of attributes (multiple response)	
Structure	133 (62.44)
Process	39 (18.31)
Outcome	41 (19.25)
Statistical models	
Logit	11 (39.29)
Latent class analysis	4 (14.29)
Multivariate	9 (32.14)
Other	4 (14.29)
Publication year	
2007-2012	4 (14.29)
2013-2018	7 (25.00)
2019-2024	17 (60.71)

### Attributes

Table 2 provides an overview of all attributes included in the identified studies. A total of 203 attributes were identified across three key dimensions: structure, process, and outcome. Overall, the studies used 129 different structure attributes, 36 process attributes, and 38 outcome attributes. The most commonly identified structure attributes were ‘cost of services’ (n = 25; reported in 25 studies), ‘distance or proximity’ (n = 24, reported in 24 studies), ‘waiting time’ (n = 13), type/qualification of care provider (n = 13), and ‘availability of medicine’ (n = 12). Among the process attributes, the most commonly identified was ‘provider’s behaviour/attitude’ (n = 19). Among the outcome attributes, the most common was ‘quality of care’ (n = 17). The predominance of structural attributes (63.5% of total) reflects the primary focus on access and affordability barriers in urban LMIC preference studies, while process and outcome attributes together comprised 36.5%, indicating that interpersonal and quality dimensions, though less frequently examined, remain important considerations.

**Table 2:** Most valued and significant attributes by preference studies

Dimensions	*n* (%)
Structure (*n* = 129)	63.55%
Distance or proximity	24 (18.6)
Appointment flexibility	2 (1.55)
Availability of medicine	12 (9.3)
Availability of provider	3 (2.33)
Availability of test/equipment	7 (5.43)
Cleanliness of the facility	3 (2.33)
Cost of services	25 (19.38)
Hospital size	1 (0.78)
Multidisciplinary care	4 (3.1)
Others	22 (17.05)
Type of care provider	13 (10.08)
Waiting time	13 (10.08)
Process (*n* = 36)	17.73%
Consultation time	4 (11.11)
Doctors’ appearance	2 (5.56)
Others	4 (11.11)
Privacy/Confidentiality	4 (11.11)
Provider’s behaviour/attitude	19 (52.78)
Relationship with provider	3 (8.33)
Outcome (*n* = 38)	18.72%
Provider reputation	6 (15.79)
Prior experience receiving services	7 (18.42)
Quality of care	17 (44.74)
Recommendation from acquaintances	6 (15.79)
Trust in provider	2 (5.26)

### Comparison between the SP and RP attributes

The comparison between attributes derived from SP and RP studies highlights the distinct insights garnered from hypothetical scenarios versus real-world behaviors within healthcare systems. We attempted to compare SP and RP attributes across the SPO domains. In terms of *structure*, the most common attributes in both SP and RP studies were distance/proximity (*n* = 12 in both), cost of services (*n* = 11 vs. *n* = 14), and waiting time (*n* = 7 vs. *n*= 6). However, RP studies encompass a broader array of attributes, such as same-gender preference and social media engagement of providers, which were absent in stated preference studies. Regarding the *process*, both types of studies prioritise provider behavior (*n* = 8 vs. *n* = 12). In terms of *outcome*, both studies emphasised quality of care (*n* = 6 vs. *n* = 11), whereas RP studies introduced additional attributes like provider reputation, recommendations from others, and trust in the provider.

**Fig. 2:**
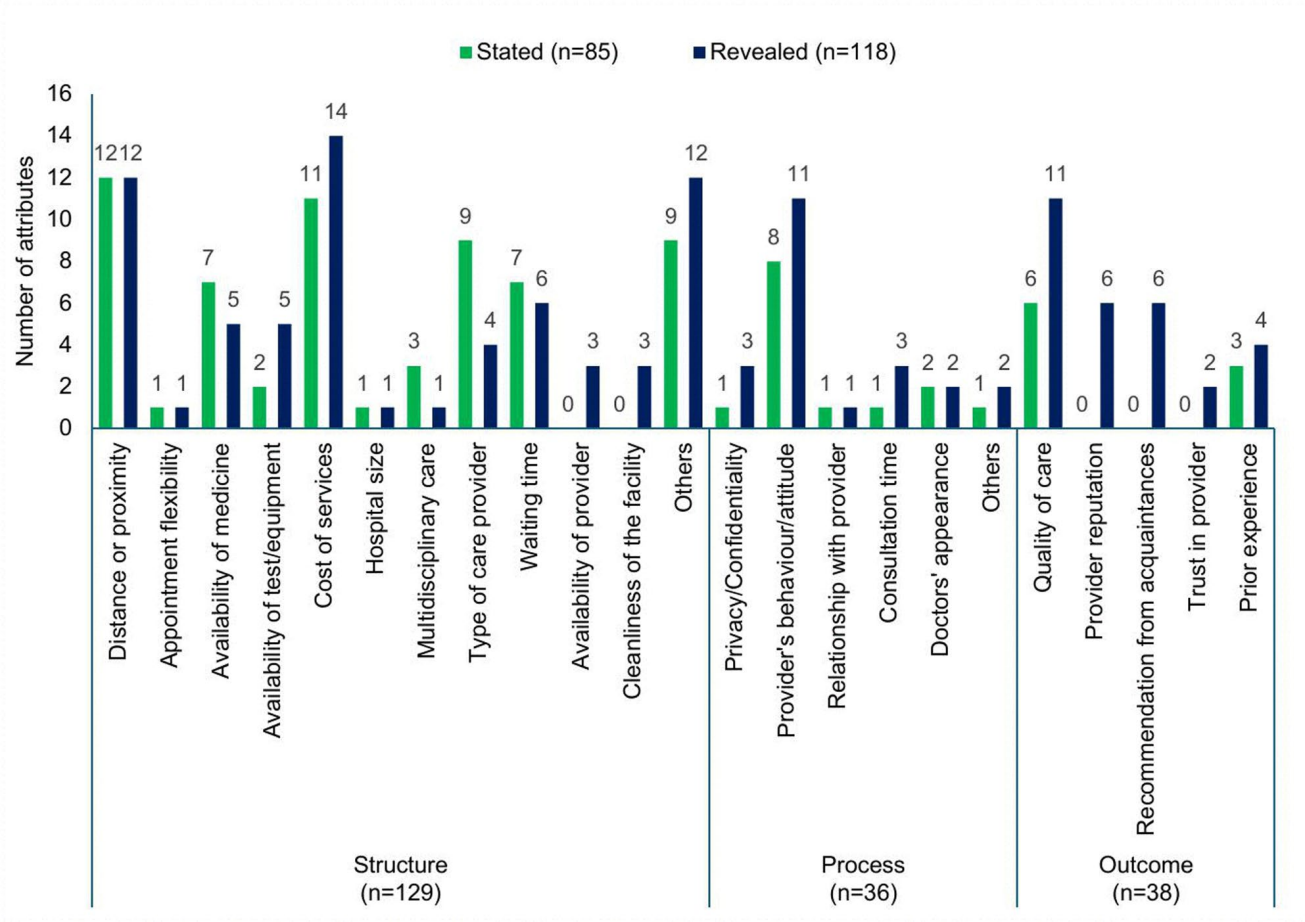
Comparison of attributes from SP and RP studies

### Most valued attributes

Based on the analysis conducted in the selected studies, we have identified the most valued/important attributes influencing preferences for PHC providers (Table 3). Two attributes consistently emerged as top priorities across both SP and RP studies: availability of medicine/equipment (most valued in 8 studies: [35, 37, 38, 41, 51, 52, 54, 56]) and distance/proximity (most valued in 6 studies: [28, 29, 33, 36, 39, 46]). Regional patterns were evident: medicine/equipment availability was particularly prominent in Sub-Saharan African studies [51, 52], while distance/proximity was emphasised in densely populated Chinese urban settings [28, 33, 36]. Beyond the core access and availability barriers, preference for specific services varied by context and population characteristics. For example, other valued attributes showed greater variation across studies, including treatment options [32, 34], waiting time [48, 57], multidisciplinary care [42], and appropriateness of care [43] were identified as the most valued attributes.

**Table 3:** Most valued and significant attributes by preference studies

Most valued attributes	SP studies	RP studies
Availability of medicine &/or equipment	(Lungu et al., 2018 [52]; Zhu et al., 2019 [38]; Jia et al., 2020 [32]; Li et al., 2021a [29]; Chiwire et al., 2022 [54]; Wang et al., 2022 [35]; Leslie et al., 2023 [44])	(Boachie, 2016 [51]; Wu et al., 2017 [37]; Jiang et al., 2020b [63])
Distance/Proximity	(Jiang et al., 2020a [63]; Peng et al., 2020 [33]; Wang et al., 2020 [36])	(Amaghionyeodiwe, 2008 [46]; Qian et al., 2010 [39]; Li et al., 2021b [64])
Quality of care	(Černauskas et al., 2018 [43])	(Qian et al., 2010 [39]; Galal and Al-Gamal, 2014 [50])
Waiting time	(Liu et al., 2020 [57]; Nguyen et al., 2023 [48])	(Sun et al., 2019 [40])
Multidisciplinary care	(Lv et al., 2023 [42])	**-**
Provider type	**-**	(Jiang et al., 2020b [63]; Khatami et al., 2020 [49])
Cost of services	**-**	(Jiang et al., 2020b [63])
Convenience of seeking healthcare	**-**	(Tang et al., 2013 [31]; Sun et al., 2019 [40])
Attitude/behaviour of the provider	**-**	(Porter and Bresick, 2017 [55])
Consultation time	**-**	(Khoo et al., 2021 [47])

### Preference heterogeneity

A total of 18 of the 28 studies have examined preference heterogeneity considering the influence of observed socioeconomic factors on the preferences of PHC providers (Appendix 4). In RP studies, education [29, 31, 37, 39, 40, 45–47, 51], age [29, 39, 40, 46, 47, 51], gender [29, 39, 45, 47, 49], income [29, 40, 46, 47, 51], and insurance status [29, 31, 39, 40, 47] were the most frequently examined heterogeneity factors. Some RP studies also examined the severity of illness [29, 37, 39, 46], employment status [29, 39, 40, 45], marital status [29, 39, 40, 45], and location of the households [31, 45, 51]. On the other hand, in SP studies, gender [28, 32, 33, 38, 42, 43, 54], age [28, 33, 38, 42, 43], education [28, 32, 33, 38, 42, 43], and severity of illness [28, 33, 42, 58] were among the frequently examined factors. Some unique dimensions, such as type of care/hospital [59], number of visits [38], family history [42], multimorbidity [42], and duration of illness [42] were also explored in SP studies. While examining the most significant factor determining preference, we found that the severity of illness was the most recurrent determinant, observed in a total of four studies [28, 37, 39, 57]. Furthermore, location emerges as a significant consideration, appearing in two studies [29, 31]. Gender [49, 54] and education [38, 43] were the most significant in two studies. Income [51], employment status [40], and ethnicity [47] were the most significant in three different studies. While considering the direction of the factors, we found severity of illness shifted preferences from access attributes such as travel or waiting time [57] towards senior or specialised providers [32] and hospital services [37, 60]. Gender differences were most evident for interpersonal attributes, with women placing greater value on provider communication [38, 61], confidentiality and shorter waiting time [54], and greater use of PHC providers in some contexts [45]. Higher education and income were associated with preferences for specialist care, modern technology, and higher-level facilities [28, 38, 62], and with lower PHC use in revealed-preference studies [29, 31, 40]. Lower income increased sensitivity to cost and reimbursement [38, 42]. Age showed mixed directions, with older individuals more likely to choose general providers in some settings [29, 31] but also expressing stronger preferences for senior providers and higher-quality care in DCEs [32, 33, 38] (Appendix 7).

### Narrative synthesis of qualitative findings

The synthesis of qualitative findings from the three included mixed-method studies [37, 49, 55] reveals a convergence around several key themes influencing patients’ preferences and experiences with healthcare services. One of the recurring themes is accessibility and convenience. Patients expressed a clear preference for healthcare facilities that were close to home or the workplace [37, 49, 55], had short waiting times [37, 49, 55], and ensured the availability of physicians and medicines [37, 49]. Affordability, including low OOP payments, was consistently highlighted across all studies as a critical factor shaping service use, especially in resource-constrained contexts [37, 49, 55].

*“It is difficult for us elderly people to walk. This [community facility] is close by.” (Wu et al. 2017)*.

The physical condition and cleanliness of facilities also influenced perceptions of care quality [37, 49, 55]. A clean and well-equipped environment contributed to feelings of safety and trust, while the availability of diagnostics and treatment options reinforced confidence in the system’s capacity to deliver effective care.

Across all three studies, participants emphasised the importance of friendly, respectful, and attentive interactions with physicians and clinic staff [37, 49, 55]. Provider expertise, qualifications, and appearance were noted to build patient trust and confidence in care quality, directly affecting satisfaction and overall experience [37, 49, 55].

*“[If you] have the freedom to choose*,* why not choose a hospital with much better resources and competent doctors?” (Wu et al. 2017)*.

Communication and interpersonal engagement also emerged as vital [37, 55]. Patients valued providers who listened, explained clearly, and used culturally familiar or faith-based language [55]. This highlights the importance of doctor-patient rapport and relational aspects of care.

*“[Community health workers] attitudes are good. It’s almost empty in the community facility but very busy in a hospital.” (Wu et al. 2017)*.

Finally, community influence played a notable role. Recommendations from acquaintances and the reputation of providers and facilities often guided the healthcare-seeking behaviour of the population [49, 55].

### Quality appraisal

The quality of the included studies that used the SP approach varied, with scores ranging from 40% to 90% across the main items of the ISPOR checklist. Among the 15 SP studies, 6 (40%) were rated as ‘average quality,’ scoring between 61–80%, whereas four studies (26.7%) were of high quality (scoring between 81–100%), and 5 studies (33.3%) were rated as low-quality, scoring 60% or less. A total of 13 RP studies, comprising a mix of quantitative and mixed-methods research designs, were appraised using MMAT. Overall, the quality of the included studies varied, with scores ranging from 71% to 100%. Among the 13 studies, 12 were categorised as ‘high quality’ with scores between 81% − 100%, and one study was of average quality (scoring 61–80%). The high-quality and average-quality studies demonstrated strengths in certain methodological aspects, such as clear descriptions of the study population and appropriate statistical analysis techniques. Whereas the low-quality study exhibited weaknesses, including inadequate reporting of the study design and sampling methods (Appendix 5 and 6).

## Discussion

### Summary of key findings

To ensure person-centred care, it is important to align the provision of PHC services with patients’ preferences, especially in low-income settings where optimising resource utilisation is crucial. In our current systematic review, we have identified, organised, and analysed a large number of attributes regarding patients’ preferences in choosing urban PHC providers, along with the factors influencing these preferences. We identified a total of 203 attributes across the 28 selected studies. The most frequently reported attributes were cost of services (*n* = 25), and distance/proximity (*n* = 24), followed closely by provider’s behaviour/attitude (*n* = 19) and quality of care (*n* = 17). In the majority of the studies, availability of medicine/equipment [35, 37, 38, 41, 51, 52, 54, 56], distance/proximity [28, 29, 33, 36, 39, 46], healthcare provider type [41, 49], and quality of care [39, 43, 50] were identified as the most valued/important attributes influencing preferences for PHC.

### Comparison of findings with existing literature

There are two other reviews on patient preferences in primary care - covering studies up to 2015 [16] and 2021 [17], whereas our review included studies until March 2024. Our review incorporated findings from 28 studies. Lim et al. [17] included 35 studies (with 16 also reported by Kleij et al.), and Kleij et al. [16] included 19 studies in their respective reviews. Notably, only two studies from the previous reviews are also included in our current analysis [36, 38]. The variation in study numbers across these reviews can be attributed to several factors. While prior reviews focused on exploring attributes of PHC preferences globally, our focus was specifically on the preferences of urban residents in LMICs for PHC providers. Despite PHC being a critical component for achieving UHC, evidence indicates that PHC systems in LMICs are underperforming [63]. Additionally, while the previous reviews [16, 17] only included outpatient PHC visits, this review covered both outpatient and inpatient PHC visits.

Furthermore, unlike the previous reviews, our scope was not limited to SP studies; instead, we covered a broader range of RP studies, including both qualitative and quantitative approaches to gather comprehensive evidence on PHC preferences, determinants of preferences, and compared the identified attributes between these two broad approaches. Including both SP and RP studies allowed us to compare the preference attributes between the stated and revealed preferences. SP studies, despite their susceptibility to biases and subjective interpretation, offer valuable insights into individuals’ expressed desires, which complement the more concrete evidence provided by revealed preferences in scholarly discussion and policy formulation [64]. We found that RP studies covered a wide range of attributes observed in patients’ actual healthcare-seeking behaviours compared to the limited number of stated attributes reported in SP studies. However, there is a pattern of similarity in the most commonly reported attributes in both SP and RP studies. Notably, RP studies identified several attributes that were under-represented or absent in SP studies, such as provider availability, same gender provider preference, cleanliness, and trustworthiness. These highlight that SP studies may underestimate factors influencing real-world healthcare choices. This also indicates that integrating insights from RP studies could improve SP design in terms of attribute relevance and external validity.

We also categorised the attributes into three domains following the Donabedian Model: Structure, Process, and Outcome. Our analysis reveals that most attributes belonged to the ‘Structure’ category, followed by ‘Outcome’ and ‘Process’ related attributes. This aligns with the findings of Kleij et al. [16], though it differs from the observations of Lim et al. [17], who identified ‘Process’ attributes as the most dominant. The differences in findings can be attributed to the different approaches used in defining the SPO and categorising the identified attributes within the reviews. This led to certain attributes, such as availability of medicine, type of provider, cost, and distance, being classified as ‘Process’ in the earlier review [17], whereas in our review, they were categorised as ‘Structure’.

The studies included by Kleij et al. [16] and the majority of those (29 out of 35) by Lim et al. [17] were conducted in high-income countries (HICs). Notably, a divergence in influencing attributes between HICs and LMICs was identified. In HICs, attributes crucial to care quality, such as providers’ qualifications [65–67], providers’ knowledge of the patient [68–70], and the level of attention provided [71–73], were identified as the most significant preference attributes. Conversely, in LMICs, the predominant influencing preference attributes revolved around the accessibility and affordability of PHC, particularly distance or proximity to healthcare facilities [28, 33, 36, 41–43, 54, 57], the cost of services [28, 36, 38, 41, 42, 57], and the availability of medicine [35, 38, 41, 42, 54]. These differences between HICs and LMICs regarding PHC preferences reflect distinct health system contexts that shape what patients prioritise. In HICs, where healthcare systems are generally well-established, pooled systems (insurance) of finance are better established and remove financial barriers. Well-established public services ensure geographic access and cover a wider range of healthcare, and strong regulation provides quality assurance, allowing patients to emphasis on aspects like quality of care. In LMICs, several factors force the prioritisation of structural attributes. Firstly weak regulation system creates fragmented provider markets and dominance of informal providers, secondly, inadequate public sector capacity drives patients to private providers, and finally, reliance on OOP expenditure for healthcare shifts priorities towards ensuring basic healthcare needs are met, such as affordability and availability of essential medicines. Urban contexts in LMICs present additional dynamics that shape these preference patterns. The dominance of informal providers in urban areas, including drug shops and unregulated clinics, creates markets, increasing reliance on cost, proximity, and resource availability as choice criteria. Moreover, many urban residents work in informal sectors with daily wage uncertainty and no sick leave, making time away from work costly and increasing the importance of proximity and short waiting times. Across the studies examined, several demographic and socioeconomic factors consistently influenced PHC provider preferences in LMICs. Some of the factors, such as age, gender, education, and severity of illness, were commonly examined in both types of studies. For example, in an SP study, Lv Y et al. [42] showed that younger patients, those with higher education, or those who were unemployed, placed less importance on travel time. They also showed that female patients paid more attention to travel time and the attitude of the medical staff. In another SP study, Liu Y et al. [58] showed that people prioritised total visit time for minor conditions, while they placed the highest importance on the equipment for severe conditions. On the other hand, in an RP study, Li X et al. [29] found that patients who are older, less educated, have lower family income, and have milder self-perceived disease status were more likely to choose primary care facilities for their first contact compared to the higher level facility. These findings on preference heterogeneity emphasise the importance of considering diverse factors when studying the preferences of PHC providers. For instance, patient preferences for different attributes varied significantly when the perceived severity of illness was taken into account. Furthermore, socioeconomic heterogeneity proves to be significant in predicting the choice of providers, and it should be considered in understanding the patient choice behaviour.

### Strengths and limitations

There are a few limitations in our review. Excluding non-English language articles may have resulted in the omission of relevant studies, although the number of full-text non-English articles was only two. Alternative search terms could have yielded different results. However, we constructed our search terms according to best practice guidelines and with expert input. We did not assess publication bias, as our review did not include a meta-analysis and the included studies were heterogeneous in terms of design, outcomes, and analytical approaches. The inherent variability in results from SPs, influenced by factors like research questions, attribute selection, and data analysis methods, prevents us from making direct comparisons. Quality appraisal revealed that five SP studies had methodological weaknesses in attribute development and analysis, though this did not systematically bias the main attributes identified.

Furthermore, the limited number of studies exploring preference heterogeneity factors did not allow us to synthesise their interactions with different attributes, which can be explored in future studies. Notably, the majority of the included studies were conducted in China, with a few in sub-Saharan African countries. Therefore, the findings are mostly context-driven and may not be fully generalisable to all urban LMIC contexts, particularly where health system structures differ substantially. Also, we acknowledge the heterogeneity across health systems in LMICs, along with the borderline of defining PHC, which should be considered when interpreting the results.

Despite these limitations, our study offers a broad synthesis of attributes that were considered when choosing PHC providers in the context of urban PHC systems in LMICs, going beyond traditional SP studies. This broader approach enables a comparison between SP and RP studies.

## Conclusion

The identification of cost, distance, and medicine availability as key attributes in this review indicates that urban PHC strengthening should prioritise: ensuring medicine availability through improved supply chains, strategic facility placement within walking distance of urban communities (particularly slum and peri-urban areas), and expanding financial protection schemes for informal sector workers. Given the dominance of informal providers in urban areas, regulatory frameworks must address quality assurance across the diverse provider landscape.

Our findings offer guidance for future preference studies on PHC providers in the LMIC context, providing a broad overview of attributes and levels. For example, future analyses could concentrate on less-studied attributes of PHC, such as the referral system and follow-up frequency. We recommend that future SP studies specify the types of illness (major or minor), as our findings suggest patients’ preferences may differ depending on the severity of illness. Additionally, we recommend exploring patient preferences across LMICs, as our review revealed a scarcity of evidence within specific country contexts, with most of the PHC preference studies concentrated in China and sub-Saharan countries. The identified attributes should be contextualised to inform policy decisions. The evidence generated in this review will help policymakers and researchers in tailoring PHC interventions to meet the specific expectations of urban communities, thereby enhancing their effectiveness, especially in urban LMIC settings. The evidence can be used to prioritise attributes based on the local context, whether as part of ongoing changes or broader health system reforms.

## Supplementary Information

Supplementary Material 1.

## Data Availability

All data generated or analysed during this study are included in this published article and in Appendix 4.

## Abbreviations

HICs: High-income countries
ISPOR: International society for pharmacoeconomics and outcomes research
LMICs: Low- and middle-income countries
MeSH: Medical subject headings
MMAT: Mixed methods appraisal tool
OOP: Out-of-pocket
PHC: Primary healthcare
PRISMA: Preferred reporting items in systematic reviews and meta-analyses
RP: Revealed preference
SP: Stated preference
UHC: Universal health coverage

## References

[1] World Health Organization. United Nations Children’s Fund. A Vision For Primary health care In The 21st century: Towards universal health coverage, and the Sustainable Development Goals. Geneva; 2018.

[2] WHO. Interim report: placing people and communities at the centre of health services: WHO global strategy on integrated people-centred health services 2016–2026: executive summary. World Health Organization; 2015.

[3] Starfield B, Shi L, Macinko J. Contribution of primary care to health systems and health. Milbank Q. 2005;83:457–502. 10.1111/j.1468-0009.2005.00409.x. 16202000 10.1111/j.1468-0009.2005.00409.x PMC2690145

[4] World Health Organization. Declaration of Alma-Ata. Alma Ata: World Health Organization. Regional Office for Europe; 1978.

[5] The Lancet Regional Health – Europe. Strengthening primary health care to achieve universal health coverage. Lancet Reg Heal - Eur. 2024;39:100897. 10.1016/j.lanepe.2024.100897. 10.1016/j.lanepe.2024.100897 PMC11129332 38803630

[6] Rasanathan K, Evans TG. Primary health care, the Declaration of Astana and COVID-19. 2020; October:801–8. 10.2471/BLT.20.252932 PMC7607474 33177777

[7] Elsey H, Agyepong I, Huque R, Quayyem Z, Baral S, Ebenso B, et al. Rethinking health systems in the context of urbanisation: challenges from four rapidly urbanising low-income and middle-income countries. BMJ Glob Heal. 2019;4:e001501. 10.1136/bmjgh-2019-001501. 10.1136/bmjgh-2019-001501 PMC6577312 31297245

[8] Khan IA, Priyanka N, Mitra SK, Lahariya AU, Vaz RP, Lahariya C. The Role of Private Practitioners in Bridging the Healthcare Gap and Achieving Universal Health Coverage in India. Prev Med Res Rev. 2024;1:260–3. 10.4103/PMRR.PMRR_26_23.

[9] Golechha M, Mavalankar D. Healthcare for All: Challenges in Urban Areas. In: Reddy KS, Vaidyanathan G, Sinha A, editors. Healthcare for All. Routledge; 2025. pp. 331–41.

[10] Rao KS. Public Health and the Role of the Private Sector. Prev Med Res Rev. 2024;1.

[11] Adams AM, Islam R, Ahmed T. Who serves the urban poor? A geospatial and descriptive analysis of health services in slum settlements in Dhaka, Bangladesh. Health Policy Plan. 2015;30(1):i32–45. 10.1093/heapol/czu094. 25759453 10.1093/heapol/czu094 PMC4353891

[12] Bigna JJ, Noubiap JJ. The rising burden of non-communicable diseases in sub-Saharan Africa. Lancet Glob Heal. 2019;7:e1295–6. 10.1016/S2214-109X(19)30370-5. 10.1016/S2214-109X(19)30370-5 31537347

[13] Ezeh A, Oyebode O, Satterthwaite D, Chen Y, Ndugwa R, Sartori J, et al. The health of people who live in slums 1 The history, geography, and sociology of slums and the health problems of people who live in slums. Lancet. 2017;389:547–58. 10.1016/S0140-6736(16)31650-6. 27760703 10.1016/S0140-6736(16)31650-6

[14] OECD. Realising the Full Potential of Primary Health Care. 2019.

[15] Lahariya C. Ayushman Bharat’ Program and Universal Health Coverage in India. Indian Pediatr. 2018;55:495–506. 10.1007/s13312-018-1341-1. 29978817

[16] Kleij KS, Tangermann U, Amelung VE, Krauth C. Patients’ preferences for primary health care - A systematic literature review of discrete choice experiments. BMC Health Serv Res. 2017;17:1–12. 10.1186/s12913-017-2433-7. 28697796 10.1186/s12913-017-2433-7 PMC5505038

[17] Lim AH, Ng SW, Teh XR, Ong SM, Sivasampu S, Lim KK. Conjoint analyses of patients’ preferences for primary care: a systematic review. BMC Prim Care. 2022;23:1–16. 10.1186/s12875-022-01822-8. 10.1186/s12875-022-01822-8 PMC9463739 36085032

[18] Montgomery MR. Urban health in low-and middle-income countries. 2015.

[19] Page MJ, McKenzie JE, Bossuyt PM, Boutron I, Hoffmann TC, Mulrow CD, et al. The PRISMA 2020 statement: an updated guideline for reporting systematic reviews. BMJ. 2021;372. 10.1136/bmj.n71. 10.1136/bmj.n71 PMC8005924 33782057

[20] Saif-Ur-Rahman KM, Mamun R, Anwar I. Identifying gaps in primary healthcare policy and governance in low-income and middle-income countries: protocol for an evidence gap map. BMJ Open. 2019;9:e024316. 10.1136/bmjopen-2018-024316. 30819705 10.1136/bmjopen-2018-024316 PMC6398635

[21] Gao Q, Prina AM, Ma Y, Aceituno D, Mayston R. Inequalities in Older age and Primary Health Care Utilization in Low- and Middle-Income Countries: A Systematic Review. Int J Heal Serv. 2022;52:99–114. 10.1177/00207314211041234. 10.1177/00207314211041234 PMC8645300 34672829

[22] Erku D, Scuffham P, Gething K, Norman R, Mekonnen AB. Stated Preference Research in Reproductive and Maternal Healthcare Services in Sub – Saharan Africa: A Systematic Review. Patient - Patient-Centered Outcomes Res. 2022;15:287–306. 10.1007/s40271-021-00553-9. 10.1007/s40271-021-00553-9 34713395

[23] Covidence systematic review software. 2014.

[24] Bridges JFP, Hauber AB, Marshall D, Lloyd A, Prosser LA, Regier DA, et al. Conjoint analysis applications in health–a checklist: a report of the ISPOR Good Research Practices for Conjoint Analysis Task Force. Value Heal J Int Soc Pharmacoeconomics Outcomes Res. 2011;14:403–13. 10.1016/j.jval.2010.11.013. 10.1016/j.jval.2010.11.013 21669364

[25] Hong QN, Fàbregues S, Bartlett G, Boardman F, Cargo M, Dagenais P, et al. The Mixed Methods Appraisal Tool (MMAT) version 2018 for information professionals and researchers. Educ Inf. 2018;34:285–91.

[26] Hong QN, Gonzalez-Reyes A, Pluye P. Improving the usefulness of a tool for appraising the quality of qualitative, quantitative and mixed methods studies, the Mixed Methods Appraisal Tool (MMAT). J Eval Clin Pract. 2018;24:459–67. 10.1111/jep.12884. 29464873 10.1111/jep.12884

[27] Donabedian A. Explorations in quality assessment and monitoring: the definition of quality and approaches to its assessment. 1980.

[28] Jiang M-Z, Fu Q, Xiong J-Y, Li X-L, Jia E-P, Peng Y-Y, et al. Preferences heterogeneity of health care utilization of community residents in China: a stated preference discrete choice experiment. BMC Health Serv Res. 2020;20:430. 10.1186/s12913-020-05134-4. 32423447 10.1186/s12913-020-05134-4 PMC7236293

[29] Li X, Zhang L, Li Z, Tang W. Patient Choice and Willingness Toward Gatekeepers as First-Contact Medical Institutions in Chinese Tiered Healthcare Delivery System: A Cross-Sectional Study. Front public Heal. 2021;9:665282. 10.3389/fpubh.2021.665282. 10.3389/fpubh.2021.665282 PMC8261039 34249837

[30] Liu J, Yin H, Zheng T, Ilia B, Wang X, Chen R, et al. Primary health institutions preference by hypertensive patients: effect of distance, trust and quality of management in the rural Heilongjiang province of China. BMC Health Serv Res. 2019;19:852. 10.1186/s12913-019-4465-7. 31747908 10.1186/s12913-019-4465-7 PMC6868842

[31] Tang C, Luo Z, Fang P, Zhang F. Do patients choose community health services (CHS) for first treatment in China? Results from a community health survey in urban areas. J Community Health. 2013;38:864–72. 10.1007/s10900-013-9691-z. 23636415 10.1007/s10900-013-9691-z

[32] Jia E, Gu Y, Peng Y, Li X, Shen X, Jiang M, et al. Preferences of patients with non-communicable diseases for primary healthcare facilities: A discrete choice experiment in Wuhan, China. Int J Environ Res Public Health. 2020;17:1–15. 10.3390/ijerph17113987. 10.3390/ijerph17113987 PMC7311994 32512772

[33] Peng Y, Jiang M, Shen X, Li X, Jia E, Xiong J. Preferences for primary healthcare services among older adults with chronic disease: A discrete choice experiment. Patient Prefer Adherence. 2020;14:1625–37. 10.2147/PPA.S265093. 32982187 10.2147/PPA.S265093 PMC7505703

[34] Li X, Jiang M, Peng Y, Shen X, Jia E, Xiong J. Community residents’ preferences for chronic disease management in Primary Care Facilities in China: a stated preference survey. Arch Public Heal. 2021;79:1–9. 10.1186/s13690-021-00728-8. 10.1186/s13690-021-00728-8 PMC8620165 34823590

[35] Wang H, Sun H, Jin C, Wang M, Luo Y, Song W, et al. Preference to Family Doctor Contracted Service of Patients with Chronic Disease in Urban China: A Discrete Choice Experiment. Patient Prefer Adherence. 2022;16:2103–14. 10.2147/PPA.S371188. PT - Journal Article. 35989974 10.2147/PPA.S371188 PMC9384844

[36] Wang X, Song K, Zhu P, Valentijn P, Huang Y, Birch S. How do type 2 diabetes patients value urban integrated primary care in China? Results of a discrete choice experiment. Int J Environ Res Public Health. 2020;17:1–12. 10.3390/ijerph17010117. 10.3390/ijerph17010117 PMC6982164 31877946

[37] Wu D, Lam TP, Lam KF, Zhou XD, Sun KS. Health reforms in china: the public’s choices for first-contact care in urban areas. Fam Pract. 2017;34:194–200. 10.1093/fampra/cmw133. 28122845 10.1093/fampra/cmw133

[38] Zhu J, Li J, Zhang Z, Li H. Patients’ choice and preference for common disease diagnosis and diabetes care: A discrete choice experiment. Int J Health Plann Manage. 2019;34:e1544–55. 10.1002/hpm.2841. 31270879 10.1002/hpm.2841

[39] Qian DF, Lucas H, Chen JY, Xu L, Zhang YG. Determinants of the use of different types of health care provider in urban China: A tracer illness study of URTI. Health Policy (New York). 2010;98:227–35. 10.1016/j.healthpol.2010.06.014. 10.1016/j.healthpol.2010.06.014 20650539

[40] Sun X, Meng H, Ye Z, Conner KO, Duan Z, Liu D. Factors associated with the choice of primary care facilities for initial treatment among rural and urban residents in Southwestern China. PLoS ONE. 2019;14:e0211984. 10.1371/journal.pone.0211984. 30730967 10.1371/journal.pone.0211984 PMC6366770

[41] Jiang S, Gu Y, Yang F, Wu T, Wang H, Cutler H, et al. Tertiary hospitals or community clinics? An enquiry into the factors affecting patients’ choice for healthcare facilities in urban China. China Econ Rev. 2020;63:101538. 10.1016/j.chieco.2020.101538. October 2019:.

[42] Lv Y, Qin J, Feng X, Li S, Tang C, Wang H. Preferences of patients with diabetes mellitus for primary healthcare institutions: a discrete choice experiment in China. BMJ Open. 2023;13:e072495. 10.1136/bmjopen-2023-072495. 37369417 10.1136/bmjopen-2023-072495 PMC10410837

[43] Černauskas V, Angeli F, Jaiswal AK, Pavlova M. Underlying determinants of health provider choice in urban slums: results from a discrete choice experiment in Ahmedabad, India. BMC Health Serv Res. 2018;18:473. 29921260 10.1186/s12913-018-3264-x PMC6006661

[44] Leslie HH, Babu GR, Dolcy Saldanha N, Turcotte-Tremblay A-M, Ravi D, Kapoor NR, et al. Population Preferences for Primary Care Models for Hypertension in Karnataka, India. JAMA Netw open. 2023;6:e232937. 10.1001/jamanetworkopen.2023.2937. 36917109 10.1001/jamanetworkopen.2023.2937 PMC10015308

[45] Abodunrin OL, Bamidele JO, Olugbenga-Bello AI, Parakoyi DB. Preferred choice of health facilities for healthcare among adult residents in Ilorin metropolis, Kwara state, Nigeria. Int J Heal Res. 2010;3:79–86. 10.4314/ijhr.v3i2.70271.

[46] Amaghionyeodiwe L. Determinants of the choice of health care provider in Nigeria. Health Care Manag Sci. 2008;11:215–27. 10.1007/s10729-007-9038-3. PT - Article. 10.1007/s10729-007-9038-3 18826000

[47] Khoo EJ, Miin LY, Yin NX, Man MK, Hui LLS, May LP et al. Family and Parental Decision Making When Choosing a Paediatric General Practice Service: What Factors Mattered Most? Child Care Pract. 2021. 10.1080/13575279.2021.1920368

[48] Nguyen HTT, Vo TQ, Tran HTB, Nguyen BT, Nguyen HT, Nguyen TD, et al. The heterogeneity of public preferences for the first healthcare visit: A discrete choice experiment in the context of Vietnam. Int J Health Plann Manage. 2023;38:473–93. 10.1002/hpm.3597. 36447363 10.1002/hpm.3597

[49] Khatami F, Shariati M, Khedmat L, Bahmani M. Patients’ preferences in selecting family physician in primary health centers: a qualitative-quantitative approach. BMC Fam Pract. 2020;21:107. 10.1186/s12875-020-01181-2. PT - Journal Article. 10.1186/s12875-020-01181-2 PMC7291526 32527224

[50] Galal SB, Al-Gamal N. Health problems and the health care provider choices: a comparative study of urban and rural households in Egypt. J Epidemiol Glob Health. 2014;4:141–9. 10.1016/j.jegh.2013.12.002 PT - Comparative Study, Journal Article, Multicenter Study, Research Support, Non-U.S. Gov’t. PMC7366373 24857182

[51] Boachie MK. Preferred primary healthcare provider choice among insured persons in Ashanti region, Ghana. Int J Heal Policy Manag. 2016;5:155–63. 10.15171/ijhpm.2015.191. 10.15171/ijhpm.2015.191 PMC4770921 26927586

[52] Lungu EA, Guda Obse A, Darker C, Biesma R. What influences where they seek care? Caregivers’ preferences for under-five child healthcare services in urban slums of Malawi: A discrete choice experiment. PLoS One. 2018;13:e0189940. 10.1371/journal.pone.0189940 PT - Journal Article, Research Support, Non-U.S. Gov’t. PMC5774690 29351299

[53] Jacobsen KH, Ansumana R, Abdirahman HA, Bockarie AS, Bangura U, Meehan KA, et al. Considerations in the selection of healthcare providers for mothers and children in Bo, Sierra Leone: reputation, cost and location. Int Health. 2012;4:307–13. 10.1016/j.inhe.2012.09.004. 24029678 10.1016/j.inhe.2012.09.004

[54] Chiwire P, Beaudart C, Evers SM, Mahomed H, Hiligsmann M. Enhancing Public Participation in Public Health Offerings: Patient Preferences for Facilities in the Western Cape Province Using a Discrete Choice Experiment. Int J Environ Res Public Health. 2022;19. 10.3390/ijerph19010590. PT - Journal Article. 10.3390/ijerph19010590 PMC8744715 35010867

[55] Porter JD, Bresick G. Is it just religious practice? Exploring patients’ reasons for choosing a faith-based primary health clinic over their local public sector primary health clinic. Afr J Prim Heal Care Fam Med. 2017;9:1–9. 10.4102/phcfm.v9i1.1219. 10.4102/phcfm.v9i1.1219 PMC5506500 28697620

[56] Leslie HH, Babu GR, Saldanha ND, Turcotte-Tremblay AM, Ravi D, Kapoor NR, et al. Population Preferences for Primary Care Models for Hypertension in Karnataka, India. JAMA Netw Open. 2023;6:1–13. 10.1001/jamanetworkopen.2023.2937. 10.1001/jamanetworkopen.2023.2937 PMC10015308 36917109

[57] Liu Y, Kong Q, Wang S, Zhong L, Van De Klundert J. The impact of hospital attributes on patient choice for first visit: Evidence from a discrete choice experiment in Shanghai, China. Health Policy Plan. 2020;35:267–78. 10.1093/heapol/czz159. 31830248 10.1093/heapol/czz159 PMC7152730

[58] Liu Y, de Bekker-Grob EW, Kong Q, Wang S, Zhong L, van de Klundert J. Preferences for health-care facilities in urban China: a discrete choice experiment. Lancet. 2018;392:S34. 10.1016/s0140-6736(18)32663-1.

[59] Chiwire P, Evers SM, Mahomed H, Hiligsmann M. Identification and Prioritization of Attributes for a Discrete Choice Experiment Using the Nominal Group Technique: Patients’ Choice of Public Health Facilities in Cape Town, South Africa. Value Heal Reg Issues. 2022;27:90–8. 10.1016/j.vhri.2021.06.005. 10.1016/j.vhri.2021.06.005 34891111

[60] Qian D, Lucas H, Chen J, Xu L, Zhang Y. Determinants of the use of different types of health care provider in urban China: a tracer illness study of URTI. Health Policy. 2010;98:227–35. 10.1016/j.healthpol.2010.06.014. 20650539 10.1016/j.healthpol.2010.06.014

[61] Khatami F, Shariati M, Khedmat L, Bahmani M. Patients’ preferences in selecting family physician in primary health centers: a qualitative-quantitative approach. BMC Fam Pract. 2020;21:107. 10.1186/s12875-020-01181-2. 32527224 10.1186/s12875-020-01181-2 PMC7291526

[62] Černauskas V, Angeli F, Jaiswal AK, Pavlova M. Underlying determinants of health provider choice in urban slums: results from a discrete choice experiment in Ahmedabad, India. BMC Health Serv Res. 2018;18:473. 10.1186/s12913-018-3264-x. 29921260 10.1186/s12913-018-3264-x PMC6006661

[63] Bitton A, Fifield J, Ratcliffe H, Karlage A, Wang H, Veillard JH, et al. Primary healthcare system performance in low-income and middle-income countries: a scoping review of the evidence from 2010 to 2017. BMJ Glob Heal. 2019;4(Suppl 8):e001551. 10.1136/bmjgh-2019-001551. 10.1136/bmjgh-2019-001551 PMC6703296 31478028

[64] de Corte K, Cairns J, Grieve R. Stated versus revealed preferences: An approach to reduce bias. Health Econ. 2021;30:1095–123. 10.1002/hec.4246. 33690931 10.1002/hec.4246

[65] Caldow J, Bond C, Ryan M, Campbell NC, Miguel FS, Kiger A, et al. Treatment of minor illness in primary care: a national survey of patient satisfaction, attitudes and preferences regarding a wider nursing role. Heal Expect. 2007;10:30–45. 10.1111/j.1369-7625.2006.00422.x. 10.1111/j.1369-7625.2006.00422.x PMC5060381 17324193

[66] Gerard K, Salisbury C, Street D, Pope C, Baxter H. Is Fast Access to General Practice all that Should Matter? A Discrete Choice Experiment of Patients’ Preferences. J Health Serv Res Policy. 2008;13 2suppl:3–10. 10.1258/jhsrp.2007.007087. 10.1258/jhsrp.2007.007087 18416923

[67] Gerard K, Lattimer V, Surridge H, George S, Turnbull J, Burgess A, et al. The introduction of integrated out-of‐hours arrangements in England: a discrete choice experiment of public preferences for alternative models of care. Heal Expect. 2006;9:60–9. 10.1111/j.1369-7625.2006.00365.x. 10.1111/j.1369-7625.2006.00365.x PMC5060322 16436162

[68] Cheraghi-Sohi S, Bower P, Mead N, McDonald R, Whalley D, Roland M. What are the key attributes of primary care for patients? Building a conceptual ‘map’ of patient preferences. Heal Expect. 2006;9:275–84. 10.1111/j.1369-7625.2006.00395.x. 10.1111/j.1369-7625.2006.00395.x PMC5060357 16911142

[69] Philips H, Mahr D, Remmen R, Weverbergh M, De Graeve D, Van Royen P. Predicting the place of out-of-hours care—A market simulation based on discrete choice analysis. Health Policy (New York). 2012;106:284–90. 10.1016/j.healthpol.2012.04.010. 10.1016/j.healthpol.2012.04.010 22595229

[70] Turner D, Tarrant C, Windridge K, Bryan S, Boulton M, Freeman G, et al. Do patients value continuity of care in general practice? An investigation using stated preference discrete choice experiments. J Health Serv Res Policy. 2007;12:132–7. 10.1258/135581907781543021. 17716414 10.1258/135581907781543021

[71] Gerard K, Tinelli M, Latter S, Blenkinsopp A, Smith A. Valuing the Extended Role of Prescribing Pharmacist in General Practice: Results from a Discrete Choice Experiment. Value Heal. 2012;15:699–707. 10.1016/j.jval.2012.02.006. 10.1016/j.jval.2012.02.006 22867779

[72] Gerard K, Tinelli M, Latter S, Smith A, Blenkinsopp A. Patients’ valuation of the prescribing nurse in primary care: a discrete choice experiment. Heal Expect. 2015;18:2223–35. 10.1111/hex.12193. 10.1111/hex.12193 PMC5810682 24720861

[73] Hole AR. Modelling heterogeneity in patients’ preferences for the attributes of a general practitioner appointment. J Health Econ. 2008;27:1078–94. 10.1016/j.jhealeco.2007.11.006. 18179837 10.1016/j.jhealeco.2007.11.006

