# Supplementary material for: Choice of primary healthcare providers among population in urban areas of low- and middle-income countries: a systematic review of literature
Source: BMC Prim Care. 2026 Mar 18;27:162. doi: 10.1186/s12875-026-03261-1 (PMC13112913; doi:10.1186/s12875-026-03261-1)
Supplement: Supplementary file 1 — Supplementary Material 1. [file 12875_2026_3261_MOESM2_ESM.docx]

**Supplementary Information**

**Appendix 1. PRISMA checklist**

| Section and Topic | Item # | Checklist item | Location where item is reported |
| --- | --- | --- | --- |
| TITLE | | |  |
| Title | 1 | Identify the report as a systematic review. |  |
| ABSTRACT | | |  |
| Abstract | 2 | See the PRISMA 2020 for Abstracts checklist. |  |
| INTRODUCTION | | |  |
| Rationale | 3 | Describe the rationale for the review in the context of existing knowledge. | 4-5 |
| Objectives | 4 | Provide an explicit statement of the objective(s) or question(s) the review addresses. | 5 |
| METHODS | | |  |
| Eligibility criteria | 5 | Specify the inclusion and exclusion criteria for the review and how studies were grouped for the syntheses. | 6 |
| Information sources | 6 | Specify all databases, registers, websites, organizations, reference lists and other sources searched or consulted to identify studies. Specify the date when each source was last searched or consulted. | 6 |
| Search strategy | 7 | Present the full search strategies for all databases, registers and websites, including any filters and limits used. | Appendix 2 |
| Selection process | 8 | Specify the methods used to decide whether a study met the inclusion criteria of the review, including how many reviewers screened each record and each report retrieved, whether they worked independently, and if applicable, details of automation tools used in the process. | 7 |
| Data collection process | 9 | Specify the methods used to collect data from reports, including how many reviewers collected data from each report, whether they worked independently, any processes for obtaining or confirming data from study investigators, and if applicable, details of automation tools used in the process. | 7-8 |
| Data items | 10a | List and define all outcomes for which data were sought. Specify whether all results that were compatible with each outcome domain in each study were sought (e.g. for all measures, time points, analyses), and if not, the methods used to decide which results to collect. | 7-8 |
|  | 10b | List and define all other variables for which data were sought (e.g. participant and intervention characteristics, funding sources). Describe any assumptions made about any missing or unclear information. | 7-8 |
| Study risk of bias assessment | 11 | Specify the methods used to assess risk of bias in the included studies, including details of the tool(s) used, how many reviewers assessed each study and whether they worked independently, and if applicable, details of automation tools used in the process. | NA |
| Effect measures | 12 | Specify for each outcome the effect measure(s) (e.g. risk ratio, mean difference) used in the synthesis or presentation of results. | NA |
| Synthesis methods | 13a | Describe the processes used to decide which studies were eligible for each synthesis (e.g. tabulating the study intervention characteristics and comparing against the planned groups for each synthesis (item #5)). | NA |
|  | 13b | Describe any methods required to prepare the data for presentation or synthesis, such as handling of missing summary statistics, or data conversions. | 9-10 |
|  | 13c | Describe any methods used to tabulate or visually display results of individual studies and syntheses. | 9-10 |
|  | 13d | Describe any methods used to synthesize results and provide a rationale for the choice(s). If meta-analysis was performed, describe the model(s), method(s) to identify the presence and extent of statistical heterogeneity, and software package(s) used. | 9-10 |
|  | 13e | Describe any methods used to explore possible causes of heterogeneity among study results (e.g. subgroup analysis, meta-regression). | NA |
|  | 13f | Describe any sensitivity analyses conducted to assess robustness of the synthesized results. | NA |
| Reporting bias assessment | 14 | Describe any methods used to assess risk of bias due to missing results in a synthesis (arising from reporting biases). | NA |
| Certainty assessment | 15 | Describe any methods used to assess certainty (or confidence) in the body of evidence for an outcome. | NA |
| RESULTS | | |  |
| Study selection | 16a | Describe the results of the search and selection process, from the number of records identified in the search to the number of studies included in the review, ideally using a flow diagram. | 11 |
|  | 16b | Cite studies that might appear to meet the inclusion criteria, but which were excluded, and explain why they were excluded. | 11 |
| Study characteristics | 17 | Cite each included study and present its characteristics. | Appendix 4 |
| Risk of bias in studies | 18 | Present assessments of risk of bias for each included study. | NA |
| Results of individual studies | 19 | For all outcomes, present, for each study: (a) summary statistics for each group (where appropriate) and (b) an effect estimates and its precision (e.g. confidence/credible interval), ideally using structured tables or plots. | Table 1, Table 2, Table 3 |
| Results of syntheses | 20a | For each synthesis, briefly summarize the characteristics and risk of bias among contributing studies. | 11-12 |
|  | 20b | Present results of all statistical syntheses conducted. If meta-analysis was done, present for each the summary estimate and its precision (e.g. confidence/credible interval) and measures of statistical heterogeneity. If comparing groups, describe the direction of the effect. | NA |
|  | 20c | Present results of all investigations of possible causes of heterogeneity among study results. | 13-14, Appendix 7 |
|  | 20d | Present results of all sensitivity analyses conducted to assess the robustness of the synthesized results. | NA |
| Reporting biases | 21 | Present assessments of risk of bias due to missing results (arising from reporting biases) for each synthesis assessed. | NA |
| Certainty of evidence | 22 | Present assessments of certainty (or confidence) in the body of evidence for each outcome assessed. | NA |
| DISCUSSION | | |  |
| Discussion | 23a | Provide a general interpretation of the results in the context of other evidence. | 17-19 |
|  | 23b | Discuss any limitations of the evidence included in the review. | 19-20 |
|  | 23c | Discuss any limitations of the review processes used. | 19-20 |
|  | 23d | Discuss implications of the results for practice, policy, and future research. | 20-21 |
| OTHER INFORMATION | | |  |
| Registration and protocol | 24a | Provide registration information for the review, including register name and registration number, or state that the review was not registered. | 5 |
|  | 24b | Indicate where the review protocol can be accessed, or state that a protocol was not prepared. | 5 |
|  | 24c | Describe and explain any amendments to information provided at registration or in the protocol. | NA |
| Support | 25 | Describe sources of financial or non-financial support for the review, and the role of the funders or sponsors in the review. | 22 |
| Competing interests | 26 | Declare any competing interests of review authors. | 22 |
| Availability of data, code and other materials | 27 | Report which of the following are publicly available and where they can be found: template data collection forms; data extracted from included studies; data used for all analyses; analytic code; any other materials used in the review. | NA |

**Appendix 2. Search Strategy**

**Global health**

| 1 | (afghan* or africa* or albania* or algeria* or angola* or antigua* or barbuda* or argentin* or armenia* or aruba* or azerbaijan* or bahrain* or bangladesh* or bengal* or bangal* or barbados* or barbadian* or bajan or bajans or belarus* or belorus* or byelarus* or byelorus* or belize* or benin* or dahomey or bhutan* or bolivia* or bosnia* or herzegovin* or botswan* or batswan* or bechuanaland* or brazil* or brasil* or bulgaria* or burkina* or burkinese* or upper volta* or burundi* or urundi* or cabo verde* or cape verde* or cambodia* or kampuchea* or khmer* or cameroon* or cameroun* or ubangi shari* or chad* or chile* or china* or chinese or colombia* or comoro* or comore* or comorian* or mayotte* or congo* or zaire* or costa rica* or "cote d'ivoir*" or "cote d' ivoir*" or cote divoir* or cote d ivoir* or ivory coast* or ivorian* or croatia* or cuba or cuban or cubans or "cuba's" or cyprus* or cypriot* or czech* or djibouti* or french somaliland* or dominica* or ecuador* or egypt* or united arab republic* or el salvador* or salvadoran* or guinea* or equatoguinea* or eritrea* or estonia* or eswatini* or swaziland* or swazi* or swati* or ethiopia* or fiji* or gabon* or gabonese* or gabonaise* or gambia* or ((georgia or georgian or georgians) not (atlanta or california or florida)) or ghana* or gibraltar* or greece* or greek* or grecian* or grenada* or grenadian* or guam* or guatemala* or guyana* or guiana* or guyanese* or haiti* or hispaniola* or hondura* or hungary* or hungarian* or india* or indonesia* or iran* or iraq* or isle of man* or jamaica* or jordan* or kazakh* or kenya* or karabati* or korea* or kosovo* or kosova* or kyrgyz* or kirgiz* or kirghiz* or laos or lao or laotian* or latvia* or lebanon* or lebanese* or lesotho* or lesothan* or lesothonian* or basutoland* or mosotho* or basotho* or liberia* or libya* or jamahiriya* or lithuania* or macedonia* or madagasca* or malagasy* or malawi* or nyasaland* or malaysia* or malay* federation or maldives* or maldivian* or indian ocean or mali or malian* or "mali's" or malta or maltese* or "malta's" or micronesia* or marshallese* or kiribati* or marshall island* or nauru or nauran or nauruans or "naurian's" or mariana or marianas or palau or paluan* or tuvalu* or mauritania* or mauritan* or mauritius* or mexico* or mexican* or moldova* or moldovia* or mongol* or montenegr* or morocco* or moroccan* or ifni or mozambique* or mozambican* or myanmar* or burma* or burmese or namibia* or nepal* or new caledonia* or netherlands antill* or nicaragua* or niger* or oman or omani or omanis or "oman's" or pakistan* or palestin* or gaza* or west bank* or panama* or paraguay* or peru or peruvian* or "peru's" or philippine* or philipine* or phillipine* or phillippine* or filipino* or filipina* or poland* or polish or pole or poles or portugal* or portuguese or puerto ric* or romania* or russia* or ussr* or soviet* or rwanda* or rwandese or ruanda* or ruandese or samoa* or navigator island* or pacific island* or polynesia* or "sao tome and principe*" or sao tomean* or santomean* or saudi arabia* or saudi or saudis or senegal* or serbia* or seychell* or sierra leone* or slovak* or sloven* or melanesia* or solomon island* or norfolk island* or somali* or sri lanka* or ceylon* or "saint kitts and nevis*" or "st kitts and nevis*" or kittian* or nevisian* or saint lucia* or st lucia* or saint vincent* or st vincent* or vincentian* or grenadine* or sudan* or surinam* or syria* or tajik* or tadjik* or tadzhik* or tanzania* or tanganyika* or thai* or timor leste* or east timor* or timorese* or togo or togoles* or "togo's" or tonga* or trinidad* or tobago* or tunisia* or turkiy* or turkey* or turk or turks or turkish or turkmen* or uganda* or ukrain* or uruguay* or uzbek* or vanuatu* or new hebrides* or venezuela* or vietnam* or viet nam* or yemen* or yugoslav* or zambia* or zimbabwe* or rhodesia* or arab* countr* or middle east* or global south or sahara* or subsahara* or magreb* or maghrib* or west indies* or caribbean* or central america* or latin america* or south america* or central asia* or north asia* or northern asia* or southeastern asia* or south eastern asia* or southeast asia* or south east asia* or west asia* or western asia* or east europe* or eastern europe* or developing countr* or developing nation* or developing population* or developing world or less developed countr* or less developed nation* or less developed world or lesser developed countr* or lesser developed nation* or lesser developed world or under developed countr* or under developed nation* or under developed world or underdeveloped countr* or underdeveloped nation* or underdeveloped world or middle income countr* or middle income nation* or middle income population* or low income countr* or low income nation* or low income population* or lower income countr* or lower income nation* or lower income population* or underserved countr* or underserved nation* or underserved population* or under served population* or under served nation* or under served population* or deprived countr* or deprived population* or high burden countr* or high burden nation* or countdown countr* or countdown nation* or poor countr* or poor nation* or poor population* or poor world or poorer countr* or poorer nation* or poorer population* or poorer world or developing econom* or less developed econom* or underdeveloped econom* or under developed econom* or middle income econom* or low income econom* or lower income econom* or low gdp or low gnp or low gross domestic or low gross national or lower gdp or lower gnp or lower gross domestic or lower gross national or lmic or lmics or third world or lami countr* or transitional countr* or emerging econom* or emerging nation*).ti,ab,hw. | 1644650 |
| --- | --- | --- |
| 2 | basic public health service*.tw. | 112 |
| 3 | (clinic or clinics).tw. | 84234 |
| 4 | (Comprehensive healthcare or Comprehensive health care).tw. | 340 |
| 5 | community health*.tw. | 27652 |
| 6 | community rehabilitation.tw. | 63 |
| 7 | exp Community Health Services/ | 5633 |
| 8 | (Essential healthcare or essential health care).tw. | 236 |
| 9 | first level hospital*.tw. | 42 |
| 10 | (General practi* or GP).tw. | 19270 |
| 11 | health centre*.tw. | 23082 |
| 12 | health station*.tw. | 148 |
| 13 | health center*.tw. | 24615 |
| 14 | healer*.tw. | 4387 |
| 15 | (primary health care or primary healthcare).tw. | 30029 |
| 16 | exp primary health care/ | 22411 |
| 17 | exp general practitioners/ | 5865 |
| 18 | healer*.tw. | 4387 |
| 19 | exp patient care/ | 43161 |
| 20 | Preventive Health*.tw. | 2162 |
| 21 | Preventive service*.tw. | 2381 |
| 22 | Primary care.tw. | 25633 |
| 23 | exp traditional healers/ | 883 |
| 24 | exp traditional medicine/ | 51031 |
| 25 | traditional medicine.tw. | 54991 |
| 26 | or/2-25 [PHC] | 269964 |
| 27 | Cities/ | 10395 |
| 28 | Ghetto*.tw. | 51 |
| 29 | informal settlement*.tw. | 815 |
| 30 | poverty areas/ | 0 |
| 31 | exp low income groups/ | 8180 |
| 32 | exp urban population/ | 2215 |
| 33 | (slum or slums).tw. | 3563 |
| 34 | (town* or city or cities or metropol*).tw. | 152251 |
| 35 | (urban* or semiurban* or semi-urban* or periurban* or peri-urban*).tw. | 137340 |
| 36 | or/27-35 [urban] | 253970 |
| 37 | choice experiment*.tw. | 1482 |
| 38 | choice model*.tw. | 271 |
| 39 | choice set*.tw. | 139 |
| 40 | conjoint analys*.tw. | 494 |
| 41 | decision making/ or exp bayesian theory/ or exp decision analysis/ | 18031 |
| 42 | decision-making.tw. | 32263 |
| 43 | (discrete choice or DCE).tw. | 1082 |
| 44 | functional measurement*.tw. | 55 |
| 45 | (maximum difference or paired comparison* or part worth utilit* or patient priorit*).tw. | 681 |
| 46 | (pairwise choice* or pair-wise choice*).tw. | 8 |
| 47 | (preference* adj1 (elicitation or based or model* or experiment* or behavio?r* or stud*)).tw. | 856 |
| 48 | Q-methodology.tw. | 106 |
| 49 | ranking.tw. | 6479 |
| 50 | revealed preference*.tw. | 82 |
| 51 | (stated choice* or stated preference*).tw. | 351 |
| 52 | willing* to pay.tw. | 4516 |
| 53 | (worst best or best worst).tw. | 203 |
| 54 | or/37-53 [Preference] | 48261 |
| 55 | 1 and 26 and 36 and 54 | 509 |
| 56 | limit 55 to english language | 466 |

**Medline**

| 1 | (afghan* or africa* or albania* or algeria* or angola* or antigua* or barbuda* or argentin* or armenia* or aruba* or azerbaijan* or bahrain* or bangladesh* or bengal* or bangal* or barbados* or barbadian* or bajan or bajans or belarus* or belorus* or byelarus* or byelorus* or belize* or benin* or dahomey or bhutan* or bolivia* or bosnia* or herzegovin* or botswan* or batswan* or bechuanaland* or brazil* or brasil* or bulgaria* or burkina* or burkinese* or upper volta* or burundi* or urundi* or cabo verde* or cape verde* or cambodia* or kampuchea* or khmer* or cameroon* or cameroun* or ubangi shari* or chad* or chile* or china* or chinese or colombia* or comoro* or comore* or comorian* or mayotte* or congo* or zaire* or costa rica* or "cote d'ivoir*" or "cote d' ivoir*" or cote divoir* or cote d ivoir* or ivory coast* or ivorian* or croatia* or cuba or cuban or cubans or "cuba's" or cyprus* or cypriot* or czech* or djibouti* or french somaliland* or dominica* or ecuador* or egypt* or united arab republic* or el salvador* or salvadoran* or guinea* or equatoguinea* or eritrea* or estonia* or eswatini* or swaziland* or swazi* or swati* or ethiopia* or fiji* or gabon* or gabonese* or gabonaise* or gambia* or ((georgia or georgian or georgians) not (atlanta or california or florida)) or ghana* or gibraltar* or greece* or greek* or grecian* or grenada* or grenadian* or guam* or guatemala* or guyana* or guiana* or guyanese* or haiti* or hispaniola* or hondura* or hungary* or hungarian* or india* or indonesia* or iran* or iraq* or isle of man* or jamaica* or jordan* or kazakh* or kenya* or karabati* or korea* or kosovo* or kosova* or kyrgyz* or kirgiz* or kirghiz* or laos or lao or laotian* or latvia* or lebanon* or lebanese* or lesotho* or lesothan* or lesothonian* or basutoland* or mosotho* or basotho* or liberia* or libya* or jamahiriya* or lithuania* or macedonia* or madagasca* or malagasy* or malawi* or nyasaland* or malaysia* or malay* federation or maldives* or maldivian* or indian ocean or mali or malian* or "mali's" or malta or maltese* or "malta's" or micronesia* or marshallese* or kiribati* or marshall island* or nauru or nauran or nauruans or "naurian's" or mariana or marianas or palau or paluan* or tuvalu* or mauritania* or mauritan* or mauritius* or mexico* or mexican* or moldova* or moldovia* or mongol* or montenegr* or morocco* or moroccan* or ifni or mozambique* or mozambican* or myanmar* or burma* or burmese or namibia* or nepal* or new caledonia* or netherlands antill* or nicaragua* or niger* or oman or omani or omanis or "oman's" or pakistan* or palestin* or gaza* or west bank* or panama* or paraguay* or peru or peruvian* or "peru's" or philippine* or philipine* or phillipine* or phillippine* or filipino* or filipina* or poland* or polish or pole or poles or portugal* or portuguese or puerto ric* or romania* or russia* or ussr* or soviet* or rwanda* or rwandese or ruanda* or ruandese or samoa* or navigator island* or pacific island* or polynesia* or "sao tome and principe*" or sao tomean* or santomean* or saudi arabia* or saudi or saudis or senegal* or serbia* or seychell* or sierra leone* or slovak* or sloven* or melanesia* or solomon island* or norfolk island* or somali* or sri lanka* or ceylon* or "saint kitts and nevis*" or "st kitts and nevis*" or kittian* or nevisian* or saint lucia* or st lucia* or saint vincent* or st vincent* or vincentian* or grenadine* or sudan* or surinam* or syria* or tajik* or tadjik* or tadzhik* or tanzania* or tanganyika* or thai* or timor leste* or east timor* or timorese* or togo or togoles* or "togo's" or tonga* or trinidad* or tobago* or tunisia* or turkiy* or turkey* or turk or turks or turkish or turkmen* or uganda* or ukrain* or uruguay* or uzbek* or vanuatu* or new hebrides* or venezuela* or vietnam* or viet nam* or yemen* or yugoslav* or zambia* or zimbabwe* or rhodesia* or arab* countr* or middle east* or global south or sahara* or subsahara* or magreb* or maghrib* or west indies* or caribbean* or central america* or latin america* or south america* or central asia* or north asia* or northern asia* or southeastern asia* or south eastern asia* or southeast asia* or south east asia* or west asia* or western asia* or east europe* or eastern europe* or developing countr* or developing nation* or developing population* or developing world or less developed countr* or less developed nation* or less developed world or lesser developed countr* or lesser developed nation* or lesser developed world or under developed countr* or under developed nation* or under developed world or underdeveloped countr* or underdeveloped nation* or underdeveloped world or middle income countr* or middle income nation* or middle income population* or low income countr* or low income nation* or low income population* or lower income countr* or lower income nation* or lower income population* or underserved countr* or underserved nation* or underserved population* or under served population* or under served nation* or under served population* or deprived countr* or deprived population* or high burden countr* or high burden nation* or countdown countr* or countdown nation* or poor countr* or poor nation* or poor population* or poor world or poorer countr* or poorer nation* or poorer population* or poorer world or developing econom* or less developed econom* or underdeveloped econom* or under developed econom* or middle income econom* or low income econom* or lower income econom* or low gdp or low gnp or low gross domestic or low gross national or lower gdp or lower gnp or lower gross domestic or lower gross national or lmic or lmics or third world or lami countr* or transitional countr* or emerging econom* or emerging nation*).ti,ab,hw,kf. | 3350265 |
| --- | --- | --- |
| 2 | basic public health service*.tw,kw. | 142 |
| 3 | (clinic or clinics).tw,kw. | 408057 |
| 4 | (Comprehensive healthcare or Comprehensive health care).tw,kw. | 1414 |
| 5 | community health*.tw,kw. | 35251 |
| 6 | community rehabilitation.tw,kw. | 587 |
| 7 | exp Community Health Services/ | 335473 |
| 8 | (Essential healthcare or essential health care).tw,kw. | 499 |
| 9 | first level hospital*.tw,kw. | 78 |
| 10 | (General practi* or GP).tw,kw. | 132198 |
| 11 | exp general practice/ | 79144 |
| 12 | health centre*.tw,kw. | 11710 |
| 13 | health station*.tw,kw. | 369 |
| 14 | health center*.tw,kw. | 28009 |
| 15 | healer*.tw,kw. | 5689 |
| 16 | (primary health care or primary healthcare).tw,kw. | 44381 |
| 17 | exp patient-centered care/ | 24699 |
| 18 | exp Primary Health Care/ | 196157 |
| 19 | Preventive Health*.tw,kw. | 6375 |
| 20 | Preventive service*.tw,kw. | 7119 |
| 21 | Primary care.tw,kw. | 151098 |
| 22 | Traditional Medicine Practitioners/ | 43 |
| 23 | traditional medicine.tw,kw. | 16223 |
| 24 | exp traditional medicine/ | 47029 |
| 25 | Cities/ | 30940 |
| 26 | Ghetto*.tw,kw. | 420 |
| 27 | informal settlement*.tw,kw. | 996 |
| 28 | poverty areas/ | 6719 |
| 29 | exp Suburban Population/ | 1971 |
| 30 | (slum or slums).tw,kw. | 4098 |
| 31 | (town* or city or cities or metropol*).tw,kw. | 264759 |
| 32 | (urban* or semiurban* or semi-urban* or periurban* or peri-urban*).tw,kw. | 220089 |
| 33 | urban hospital/ | 7826 |
| 34 | urban health/ | 18355 |
| 35 | urban population/ | 63045 |
| 36 | choice experiment*.tw,kw. | 4698 |
| 37 | choice model*.tw,kw. | 964 |
| 38 | choice set*.tw,kw. | 587 |
| 39 | conjoint analys*.tw,kw. | 1240 |
| 40 | exp Choice Behavior/ | 61617 |
| 41 | decision-making.tw,kw. | 207083 |
| 42 | (discrete choice or DCE).tw,kw. | 10436 |
| 43 | functional measurement*.tw,kw. | 1214 |
| 44 | maximum difference.tw,kw. | 1706 |
| 45 | paired comparison*.tw,kw. | 2908 |
| 46 | part worth utilit*.tw,kw. | 59 |
| 47 | patient priorit*.tw,kw. | 739 |
| 48 | Patient Preference/ | 10903 |
| 49 | (pairwise choice* or pair-wise choice*).tw,kw. | 72 |
| 50 | (preference* adj1 (elicitation or based or model* or experiment* or behavio?r* or stud*)).tw,kw. | 5587 |
| 51 | Q-methodology.tw,kw. | 681 |
| 52 | ranking.tw,kw. | 34109 |
| 53 | revealed preference*.tw,kw. | 263 |
| 54 | (stated choice* or stated preference*).tw,kw. | 1192 |
| 55 | willing* to pay.tw,kw. | 10418 |
| 56 | (worst best or best worst).tw,kw. | 731 |
| 57 | or/2-24 [PHC] | 1209216 |
| 58 | or/25-35 [Urban] | 477324 |
| 59 | or/36-56 [Preference] | 335217 |
| 60 | 1 and 57 and 58 and 59 | 1025 |
| 61 | limit 60 to english language | 972 |

**Embase**

| 1 | (afghan* or africa* or albania* or algeria* or angola* or antigua* or barbuda* or argentin* or armenia* or aruba* or azerbaijan* or bahrain* or bangladesh* or bengal* or bangal* or barbados* or barbadian* or bajan or bajans or belarus* or belorus* or byelarus* or byelorus* or belize* or benin* or dahomey or bhutan* or bolivia* or bosnia* or herzegovin* or botswan* or batswan* or bechuanaland* or brazil* or brasil* or bulgaria* or burkina* or burkinese* or upper volta* or burundi* or urundi* or cabo verde* or cape verde* or cambodia* or kampuchea* or khmer* or cameroon* or cameroun* or ubangi shari* or chad* or chile* or china* or chinese or colombia* or comoro* or comore* or comorian* or mayotte* or congo* or zaire* or costa rica* or "cote d'ivoir*" or "cote d' ivoir*" or cote divoir* or cote d ivoir* or ivory coast* or ivorian* or croatia* or cuba or cuban or cubans or "cuba's" or cyprus* or cypriot* or czech* or djibouti* or french somaliland* or dominica* or ecuador* or egypt* or united arab republic* or el salvador* or salvadoran* or guinea* or equatoguinea* or eritrea* or estonia* or eswatini* or swaziland* or swazi* or swati* or ethiopia* or fiji* or gabon* or gabonese* or gabonaise* or gambia* or ((georgia or georgian or georgians) not (atlanta or california or florida)) or ghana* or gibraltar* or greece* or greek* or grecian* or grenada* or grenadian* or guam* or guatemala* or guyana* or guiana* or guyanese* or haiti* or hispaniola* or hondura* or hungary* or hungarian* or india* or indonesia* or iran* or iraq* or isle of man* or jamaica* or jordan* or kazakh* or kenya* or karabati* or korea* or kosovo* or kosova* or kyrgyz* or kirgiz* or kirghiz* or laos or lao or laotian* or latvia* or lebanon* or lebanese* or lesotho* or lesothan* or lesothonian* or basutoland* or mosotho* or basotho* or liberia* or libya* or jamahiriya* or lithuania* or macedonia* or madagasca* or malagasy* or malawi* or nyasaland* or malaysia* or malay* federation or maldives* or maldivian* or indian ocean or mali or malian* or "mali's" or malta or maltese* or "malta's" or micronesia* or marshallese* or kiribati* or marshall island* or nauru or nauran or nauruans or "naurian's" or mariana or marianas or palau or paluan* or tuvalu* or mauritania* or mauritan* or mauritius* or mexico* or mexican* or moldova* or moldovia* or mongol* or montenegr* or morocco* or moroccan* or ifni or mozambique* or mozambican* or myanmar* or burma* or burmese or namibia* or nepal* or new caledonia* or netherlands antill* or nicaragua* or niger* or oman or omani or omanis or "oman's" or pakistan* or palestin* or gaza* or west bank* or panama* or paraguay* or peru or peruvian* or "peru's" or philippine* or philipine* or phillipine* or phillippine* or filipino* or filipina* or poland* or polish or pole or poles or portugal* or portuguese or puerto ric* or romania* or russia* or ussr* or soviet* or rwanda* or rwandese or ruanda* or ruandese or samoa* or navigator island* or pacific island* or polynesia* or "sao tome and principe*" or sao tomean* or santomean* or saudi arabia* or saudi or saudis or senegal* or serbia* or seychell* or sierra leone* or slovak* or sloven* or melanesia* or solomon island* or norfolk island* or somali* or sri lanka* or ceylon* or "saint kitts and nevis*" or "st kitts and nevis*" or kittian* or nevisian* or saint lucia* or st lucia* or saint vincent* or st vincent* or vincentian* or grenadine* or sudan* or surinam* or syria* or tajik* or tadjik* or tadzhik* or tanzania* or tanganyika* or thai* or timor leste* or east timor* or timorese* or togo or togoles* or "togo's" or tonga* or trinidad* or tobago* or tunisia* or turkiy* or turkey* or turk or turks or turkish or turkmen* or uganda* or ukrain* or uruguay* or uzbek* or vanuatu* or new hebrides* or venezuela* or vietnam* or viet nam* or yemen* or yugoslav* or zambia* or zimbabwe* or rhodesia* or arab* countr* or middle east* or global south or sahara* or subsahara* or magreb* or maghrib* or west indies* or caribbean* or central america* or latin america* or south america* or central asia* or north asia* or northern asia* or southeastern asia* or south eastern asia* or southeast asia* or south east asia* or west asia* or western asia* or east europe* or eastern europe* or developing countr* or developing nation* or developing population* or developing world or less developed countr* or less developed nation* or less developed world or lesser developed countr* or lesser developed nation* or lesser developed world or under developed countr* or under developed nation* or under developed world or underdeveloped countr* or underdeveloped nation* or underdeveloped world or middle income countr* or middle income nation* or middle income population* or low income countr* or low income nation* or low income population* or lower income countr* or lower income nation* or lower income population* or underserved countr* or underserved nation* or underserved population* or under served population* or under served nation* or under served population* or deprived countr* or deprived population* or high burden countr* or high burden nation* or countdown countr* or countdown nation* or poor countr* or poor nation* or poor population* or poor world or poorer countr* or poorer nation* or poorer population* or poorer world or developing econom* or less developed econom* or underdeveloped econom* or under developed econom* or middle income econom* or low income econom* or lower income econom* or low gdp or low gnp or low gross domestic or low gross national or lower gdp or lower gnp or lower gross domestic or lower gross national or lmic or lmics or third world or lami countr* or transitional countr* or emerging econom* or emerging nation*).ti,ab,hw,kf. | 4291060 |
| --- | --- | --- |
| 2 | choice experiment*.tw,kw. | 6034 |
| 3 | choice model*.tw,kw. | 1034 |
| 4 | choice set*.tw,kw. | 800 |
| 5 | conjoint analys*.tw,kw. | 1596 |
| 6 | exp decision making/ | 490784 |
| 7 | decision-making.tw,kw. | 284526 |
| 8 | (discrete choice or DCE).tw,kw. | 15221 |
| 9 | functional measurement*.tw,kw. | 1787 |
| 10 | maximum difference.tw,kw. | 2533 |
| 11 | paired comparison*.tw,kw. | 4085 |
| 12 | part worth utilit*.tw,kw. | 104 |
| 13 | patient priorit*.tw,kw. | 1131 |
| 14 | Patient Preference/ | 27998 |
| 15 | (pairwise choice* or pair-wise choice*).tw,kw. | 82 |
| 16 | (preference* adj1 (elicitation or based or model* or experiment* or behavio?r* or stud*)).tw,kw. | 7242 |
| 17 | Q-methodology.tw,kw. | 763 |
| 18 | ranking.tw,kw. | 42906 |
| 19 | revealed preference*.tw,kw. | 290 |
| 20 | stated choice*.tw,kw. | 134 |
| 21 | willing* to pay.tw,kw. | 15498 |
| 22 | (worst best or best worst).tw,kw. | 1001 |
| 23 | Cities/ | 56517 |
| 24 | Ghetto*.tw,kw. | 531 |
| 25 | informal settlement*.tw,kw. | 1011 |
| 26 | poverty areas/ | 58754 |
| 27 | exp Suburban Population/ | 973 |
| 28 | (slum or slums).tw,kw. | 4794 |
| 29 | (town* or city or cities or metropol*).tw,kw. | 355408 |
| 30 | (urban* or semiurban* or semi-urban* or periurban* or peri-urban*).tw,kw. | 283038 |
| 31 | urban hospital/ | 3134 |
| 32 | urban health/ | 1827 |
| 33 | urban population/ | 55452 |
| 34 | urban area/ | 83632 |
| 35 | basic public health service*.tw,kw. | 155 |
| 36 | (clinic or clinics).tw,kw. | 707080 |
| 37 | (Comprehensive healthcare or Comprehensive health care).tw,kw. | 1945 |
| 38 | community health*.tw,kw. | 43531 |
| 39 | community rehabilitation.tw,kw. | 1075 |
| 40 | exp community care/ | 143994 |
| 41 | (Essential healthcare or essential health care).tw,kw. | 558 |
| 42 | first level hospital*.tw,kw. | 118 |
| 43 | (General practi* or GP).tw,kw. | 184220 |
| 44 | exp general practice/ | 93933 |
| 45 | health centre*.tw,kw. | 15740 |
| 46 | health station*.tw,kw. | 403 |
| 47 | health center*.tw,kw. | 37455 |
| 48 | healer*.tw,kw. | 7332 |
| 49 | (primary health care or primary healthcare).tw,kw. | 50444 |
| 50 | exp patient-centered care/ | 1067890 |
| 51 | exp Primary Health Care/ | 216521 |
| 52 | Preventive Health*.tw,kw. | 7937 |
| 53 | Preventive service*.tw,kw. | 8778 |
| 54 | Primary care.tw,kw. | 208273 |
| 55 | population-based intervention*.tw,kw. | 608 |
| 56 | traditional healer/ | 2207 |
| 57 | traditional medicine.tw,kw. | 24759 |
| 58 | exp traditional medicine/ | 145941 |
| 59 | or/2-22 [preference] | 693350 |
| 60 | or/23-34 [urban] | 666867 |
| 61 | or/35-58 [PHC] | 2418396 |
| 62 | 1 and 59 and 60 and 61 | 1575 |
| 63 | limit 62 to english language | 1536 |

**APA PsycInfo**

| 1 | basic public health service*.tw. | 13 |
| --- | --- | --- |
| 2 | (clinic or clinics).tw. | 80017 |
| 3 | (Comprehensive healthcare or Comprehensive health care).tw. | 330 |
| 4 | community health*.tw. | 9734 |
| 5 | community rehabilitation.tw. | 452 |
| 6 | exp Community Health/ | 8702 |
| 7 | (Essential healthcare or essential health care).tw. | 82 |
| 8 | first level hospital*.tw. | 4 |
| 9 | (General practi* or GP).tw. | 17309 |
| 10 | exp General Practitioners/ | 6525 |
| 11 | health centre*.tw. | 1550 |
| 12 | health station*.tw. | 21 |
| 13 | health center*.tw. | 10327 |
| 14 | healer*.tw. | 3212 |
| 15 | (primary health care or primary healthcare).tw. | 8986 |
| 16 | exp patient-centered care/ | 873 |
| 17 | exp Primary Health Care/ | 22211 |
| 18 | Preventive Health*.tw. | 2018 |
| 19 | Preventive service*.tw. | 1709 |
| 20 | Primary care.tw. | 37362 |
| 21 | Traditional Medicine Practitioners/ | 0 |
| 22 | traditional medicine.tw. | 792 |
| 23 | 1 or 2 or 3 or 4 or 5 or 6 or 7 or 8 or 9 or 10 or 11 or 12 or 13 or 14 or 15 or 16 or 17 or 18 or 19 or 20 or 21 or 22 | 158258 |
| 24 | exp Urban Environments/ | 30494 |
| 25 | Cities.tw. | 18070 |
| 26 | Ghetto*.tw. | 961 |
| 27 | informal settlement*.tw. | 273 |
| 28 | exp Poverty Areas/ | 774 |
| 29 | Suburban Population.tw. | 36 |
| 30 | (slum or slums).tw. | 1127 |
| 31 | (town* or city or cities or metropol*).tw. | 88674 |
| 32 | (urban* or semiurban* or semi-urban* or periurban* or peri-urban*).tw. | 77879 |
| 33 | exp urban health/ | 316 |
| 34 | urban hospital.tw. | 493 |
| 35 | urban population.tw. | 973 |
| 36 | or/24-35 [urban] | 155039 |
| 37 | choice experiment*.tw. | 2147 |
| 38 | choice model*.tw. | 1845 |
| 39 | choice set*.tw. | 879 |
| 40 | conjoint analys*.tw. | 862 |
| 41 | exp Choice Behavior/ | 59268 |
| 42 | decision-making.tw. | 122899 |
| 43 | (discrete choice or DCE).tw. | 1691 |
| 44 | functional measurement*.tw. | 283 |
| 45 | maximum difference.tw. | 89 |
| 46 | paired comparison*.tw. | 2049 |
| 47 | part worth utilit*.tw. | 34 |
| 48 | patient priorit*.tw. | 113 |
| 49 | (pairwise choice* or pair-wise choice*).tw. | 74 |
| 50 | (preference* adj1 (elicitation or based or model* or experiment* or behavio?r* or stud*)).tw. | 4743 |
| 51 | Q-methodology.tw. | 899 |
| 52 | ranking.tw. | 10229 |
| 53 | revealed preference*.tw. | 381 |
| 54 | (stated choice* or stated preference*).tw. | 858 |
| 55 | willing* to pay.tw. | 3291 |
| 56 | (worst best or best worst).tw. | 288 |
| 57 | Patient Preference.tw. | 827 |
| 58 | or/37-57 [preferenc] | 197579 |
| 59 | (afghan* or africa* or albania* or algeria* or angola* or antigua* or barbuda* or argentin* or armenia* or aruba* or azerbaijan* or bahrain* or bangladesh* or bengal* or bangal* or barbados* or barbadian* or bajan or bajans or belarus* or belorus* or byelarus* or byelorus* or belize* or benin* or dahomey or bhutan* or bolivia* or bosnia* or herzegovin* or botswan* or batswan* or bechuanaland* or brazil* or brasil* or bulgaria* or burkina* or burkinese* or upper volta* or burundi* or urundi* or cabo verde* or cape verde* or cambodia* or kampuchea* or khmer* or cameroon* or cameroun* or ubangi shari* or chad* or chile* or china* or chinese or colombia* or comoro* or comore* or comorian* or mayotte* or congo* or zaire* or costa rica* or "cote d'ivoir*" or "cote d' ivoir*" or cote divoir* or cote d ivoir* or ivory coast* or ivorian* or croatia* or cuba or cuban or cubans or "cuba's" or cyprus* or cypriot* or czech* or djibouti* or french somaliland* or dominica* or ecuador* or egypt* or united arab republic* or el salvador* or salvadoran* or guinea* or equatoguinea* or eritrea* or estonia* or eswatini* or swaziland* or swazi* or swati* or ethiopia* or fiji* or gabon* or gabonese* or gabonaise* or gambia* or ((georgia or georgian or georgians) not (atlanta or california or florida)) or ghana* or gibraltar* or greece* or greek* or grecian* or grenada* or grenadian* or guam* or guatemala* or guyana* or guiana* or guyanese* or haiti* or hispaniola* or hondura* or hungary* or hungarian* or india* or indonesia* or iran* or iraq* or isle of man* or jamaica* or jordan* or kazakh* or kenya* or karabati* or korea* or kosovo* or kosova* or kyrgyz* or kirgiz* or kirghiz* or laos or lao or laotian* or latvia* or lebanon* or lebanese* or lesotho* or lesothan* or lesothonian* or basutoland* or mosotho* or basotho* or liberia* or libya* or jamahiriya* or lithuania* or macedonia* or madagasca* or malagasy* or malawi* or nyasaland* or malaysia* or malay* federation or maldives* or maldivian* or indian ocean or mali or malian* or "mali's" or malta or maltese* or "malta's" or micronesia* or marshallese* or kiribati* or marshall island* or nauru or nauran or nauruans or "naurian's" or mariana or marianas or palau or paluan* or tuvalu* or mauritania* or mauritan* or mauritius* or mexico* or mexican* or moldova* or moldovia* or mongol* or montenegr* or morocco* or moroccan* or ifni or mozambique* or mozambican* or myanmar* or burma* or burmese or namibia* or nepal* or new caledonia* or netherlands antill* or nicaragua* or niger* or oman or omani or omanis or "oman's" or pakistan* or palestin* or gaza* or west bank* or panama* or paraguay* or peru or peruvian* or "peru's" or philippine* or philipine* or phillipine* or phillippine* or filipino* or filipina* or poland* or polish or pole or poles or portugal* or portuguese or puerto ric* or romania* or russia* or ussr* or soviet* or rwanda* or rwandese or ruanda* or ruandese or samoa* or navigator island* or pacific island* or polynesia* or "sao tome and principe*" or sao tomean* or santomean* or saudi arabia* or saudi or saudis or senegal* or serbia* or seychell* or sierra leone* or slovak* or sloven* or melanesia* or solomon island* or norfolk island* or somali* or sri lanka* or ceylon* or "saint kitts and nevis*" or "st kitts and nevis*" or kittian* or nevisian* or saint lucia* or st lucia* or saint vincent* or st vincent* or vincentian* or grenadine* or sudan* or surinam* or syria* or tajik* or tadjik* or tadzhik* or tanzania* or tanganyika* or thai* or timor leste* or east timor* or timorese* or togo or togoles* or "togo's" or tonga* or trinidad* or tobago* or tunisia* or turkiy* or turkey* or turk or turks or turkish or turkmen* or uganda* or ukrain* or uruguay* or uzbek* or vanuatu* or new hebrides* or venezuela* or vietnam* or viet nam* or yemen* or yugoslav* or zambia* or zimbabwe* or rhodesia* or arab* countr* or middle east* or global south or sahara* or subsahara* or magreb* or maghrib* or west indies* or caribbean* or central america* or latin america* or south america* or central asia* or north asia* or northern asia* or southeastern asia* or south eastern asia* or southeast asia* or south east asia* or west asia* or western asia* or east europe* or eastern europe* or developing countr* or developing nation* or developing population* or developing world or less developed countr* or less developed nation* or less developed world or lesser developed countr* or lesser developed nation* or lesser developed world or under developed countr* or under developed nation* or under developed world or underdeveloped countr* or underdeveloped nation* or underdeveloped world or middle income countr* or middle income nation* or middle income population* or low income countr* or low income nation* or low income population* or lower income countr* or lower income nation* or lower income population* or underserved countr* or underserved nation* or underserved population* or under served population* or under served nation* or under served population* or deprived countr* or deprived population* or high burden countr* or high burden nation* or countdown countr* or countdown nation* or poor countr* or poor nation* or poor population* or poor world or poorer countr* or poorer nation* or poorer population* or poorer world or developing econom* or less developed econom* or underdeveloped econom* or under developed econom* or middle income econom* or low income econom* or lower income econom* or low gdp or low gnp or low gross domestic or low gross national or lower gdp or lower gnp or lower gross domestic or lower gross national or lmic or lmics or third world or lami countr* or transitional countr* or emerging econom* or emerging nation*).ti,ab,tw. | 549537 |
| 60 | 23 and 36 and 58 and 59 [all combined] | 150 |
| 61 | limit 60 to english language | 149 |

**Scopus**

| ( ( PUBYEAR > 1946 AND PUBYEAR < 2024 ) OR ( PUBDATETXT ( january 2024 ) OR PUBDATETXT ( february 2024 ) OR PUBDATETXT ( march 2024 ) ) ) AND ( ( TITLE-ABS-KEY ( cities OR ghetto* OR "informal settlement*" OR "poverty areas" OR "Suburban Population" OR slum OR slums OR town* OR city OR cities OR metropol* OR urban* OR semiurban* OR semi-urban* OR periurban* OR peri-urban* OR "urban hospital" OR "urban health" OR "urban population" ) ) AND ( TITLE-ABS-KEY ( "choice experiment*" OR "choice model*" OR "choice set*" OR "conjoint analys*" OR "Choice Behavior" OR "decision-making" OR "discrete choice" OR dce OR "functional measurement*" OR "maximum difference" OR "paired comparison*" OR "part worth utilit*" OR "patient priorit*" OR "Patient Preference" OR "pairwise choice*" OR "pair-wise choice*" OR "preference* elicitation" OR "preference based model*" OR "preference experiment*" OR "preference behavio?r*" OR "preference based stud*" OR q-methodology OR ranking OR "revealed preference*" OR "stated choice*" OR "stated preference*" OR "willing* to pay" OR "worst best" OR "best worst" ) ) AND ( TITLE-ABS-KEY ( afghan* OR africa* OR albania* OR algeria* OR angola* OR antigua* OR barbuda* OR argentin* OR armenia* OR aruba* OR azerbaijan* OR bahrain* OR bangladesh* OR bengal* OR bangal* OR barbados* OR barbadian* OR bajan OR bajans OR belarus* OR belorus* OR byelarus* OR byelorus* OR belize* OR benin* OR dahomey OR bhutan* OR bolivia* OR bosnia* OR herzegovin* OR botswan* OR batswan* OR bechuanaland* OR brazil* OR brasil* OR bulgaria* OR burkina* OR burkinese* OR "upper volta*" OR burundi* OR urundi* OR "cabo verde*" OR "cape verde*" OR cambodia* OR kampuchea* OR khmer* OR cameroon* OR cameroun* OR "ubangi shari*" OR chad* OR chile* OR china* OR chinese OR colombia* OR comoro* OR comore* OR comorian* OR mayotte* OR congo* OR zaire* OR "costa rica*" OR "cote d&apos;ivoir*" OR "cote d&apos; ivoir*" OR "cote divoir*" OR "cote d ivoir*" OR "ivory coast*" OR ivorian* OR croatia* OR cuba OR cuban OR cubans OR "cuba&apos;s" OR cyprus* OR cypriot* OR czech* OR djibouti* OR "french somaliland*" OR dominica* OR ecuador* OR egypt* OR "united arab republic*" OR "el salvador*" OR salvadoran* OR guinea* OR equatoguinea* OR eritrea* OR estonia* OR eswatini* OR swaziland* OR swazi* OR swati* OR ethiopia* OR fiji* OR gabon* OR gabonese* OR gabonaise* OR gambia* OR ( ( georgia OR georgian OR georgians ) AND NOT ( atlanta OR california OR florida ) ) OR ghana* OR gibraltar* OR greece* OR greek* OR grecian* OR grenada* OR grenadian* OR guam* OR guatemala* OR guyana* OR guiana* OR guyanese* OR haiti* OR hispaniola* OR hondura* OR hungary* OR hungarian* OR india* OR indonesia* OR iran* OR iraq* OR "isle of man*" OR jamaica* OR jordan* OR kazakh* OR kenya* OR karabati* OR korea* OR kosovo* OR kosova* OR kyrgyz* OR kirgiz* OR kirghiz* OR laos OR lao OR laotian* OR latvia* OR lebanon* OR lebanese* OR lesotho* OR lesothan* OR lesothonian* OR basutoland* OR mosotho* OR basotho* OR liberia* OR libya* OR jamahiriya* OR lithuania* OR macedonia* OR madagasca* OR malagasy* OR malawi* OR nyasaland* OR malaysia* OR "malay federation" OR maldives* OR maldivian* OR "indian ocean" OR mali OR malian* OR "mali&apos;s" OR malta OR maltese* OR "malta&apos;s" OR micronesia* OR marshallese* OR kiribati* OR "marshall island*" OR nauru OR nauran OR nauruans OR "naurian&apos;s" OR mariana OR marianas OR palau OR paluan* OR tuvalu* OR mauritania* OR mauritan* OR mauritius* OR mexico* OR mexican* OR moldova* OR moldovia* OR mongol* OR montenegr* OR morocco* OR moroccan* OR ifni OR mozambique* OR mozambican* OR myanmar* OR burma* OR burmese OR namibia* OR nepal* OR "new caledonia*" OR "netherlands antill*" OR nicaragua* OR niger* OR oman OR omani OR omanis OR "oman&apos;s" OR pakistan* OR palestin* OR gaza* OR "west bank*" OR panama* OR paraguay* OR peru OR peruvian* OR "peru&apos;s" OR philippine* OR philipine* OR phillipine* OR phillippine* OR filipino* OR filipina* OR poland* OR polish OR pole OR poles OR portugal* OR portuguese OR "puerto ric*" OR romania* OR russia* OR ussr* OR soviet* OR rwanda* OR rwandese OR ruanda* OR ruandese OR samoa* OR "navigator island*" OR "pacific island*" OR polynesia* OR "sao tome and principe*" OR "sao tomean*" OR santomean* OR "saudi arabia*" OR saudi OR saudis OR senegal* OR serbia* OR seychell* OR "sierra leone*" OR slovak* OR sloven* OR melanesia* OR "solomon island*" OR "norfolk island*" OR somali* OR "sri lanka*" OR ceylon* OR "saint kitts and nevis*" OR "st kitts and nevis*" OR kittian* OR nevisian* OR "saint lucia*" OR "st lucia*" OR "saint vincent*" OR "st vincent*" OR vincentian* OR grenadine* OR sudan* OR surinam* OR syria* OR tajik* OR tadjik* OR tadzhik* OR tanzania* OR tanganyika* OR thai* OR "timor leste*" OR "east timor*" OR timorese* OR togo OR togoles* OR "togo&apos;s" OR tonga* OR trinidad* OR tobago* OR tunisia* OR turkiy* OR turkey* OR turk OR turks OR turkish OR turkmen* OR uganda* OR ukrain* OR uruguay* OR uzbek* OR vanuatu* OR "new hebrides*" OR venezuela* OR vietnam* OR "viet nam*" OR yemen* OR yugoslav* OR zambia* OR zimbabwe* OR rhodesia* OR "arab countr*" OR "middle east*" OR "global south" OR sahara* OR subsahara* OR magreb* OR maghrib* OR "west indies*" OR caribbean* OR "central america*" OR "latin america*" OR "south america*" OR "central asia*" OR "north asia*" OR "northern asia*" OR "southeastern asia*" OR "south eastern asia*" OR "southeast asia*" OR "south east asia*" OR "west asia*" OR "western asia*" OR "east europe*" OR "eastern europe*" OR "developing countr*" OR "developing nation*" OR "developing population*" OR "developing world" OR "less developed countr*" OR "less developed nation*" OR "less developed world" OR "lesser developed countr*" OR "lesser developed nation*" OR "lesser developed world" OR "under developed countr*" OR "under developed nation*" OR "under developed world" OR "underdeveloped countr*" OR "underdeveloped nation*" OR "underdeveloped world" OR "middle income countr*" OR "middle income nation*" OR "middle income population*" OR "low income countr*" OR "low income nation*" OR "low income population*" OR "lower income countr*" OR "lower income nation*" OR "lower income population*" OR "underserved countr*" OR "underserved nation*" OR "underserved population*" OR "under served population*" OR "under served nation*" OR "under served population*" OR "deprived countr*" OR "deprived population*" OR "high burden countr*" OR "high burden nation*" OR "countdown countr*" OR "countdown nation*" OR "poor countr*" OR "poor nation*" OR "poor population*" OR "poor world" OR "poorer countr*" OR "poorer nation*" OR "poorer population*" OR "poorer world" OR "developing econom" OR "less developed econom*" OR "underdeveloped econom*" OR "under developed econom*" OR "middle income econom*" OR "low income econom*" OR "lower income econom*" OR "low gdp" OR "low gnp" OR "low gross domestic" OR "low gross national" OR "lower gdp" OR "lower gnp" OR "lower gross domestic" OR "lower gross national" OR lmic OR lmics OR "third world" OR "lami countr*" OR "transitional countr*" OR "emerging econom*" OR "emerging nation*" ) ) AND ( TITLE-ABS-KEY ( "basic public health service*" OR clinic OR clinics OR "Comprehensive healthcare" OR "Comprehensive health care" OR "community health*" OR "community rehabilitation" OR "Community Health Service*" OR "Essential healthcare" OR "essential health care" OR "first level hospital*" OR "General practi*" OR gp OR "general practice" OR "health centre*" OR "health station*" OR "health center*" OR healer* OR "primary health care" OR "primary healthcare" OR "patient-centered care" OR "Primary Health Care" OR "Preventive Health*" OR "Preventive service*" OR "Primary care" OR "Traditional Medicine Practitioners" OR "traditional medicine" ) ) ) AND ( LIMIT-TO ( LANGUAGE , "English" ) ) | 1225 |
| --- | --- |

**Appendix 3. Inclusion and exclusion criteria**

**Population**

- Primary studies conducted among general participants aged 18 years and older
- Participants shared their preferences related to health conditions requiring PHC, irrespective of disease types

**Comparator**

- No specific comparison criteria are set for this systematic review, as the focus will be on identifying the attributes that influence the choice of PHC providers, making direct comparisons between groups or interventions, are not applicable

**Outcome**

- Studies that report people’s preference attributes, such as distance to healthcare facility, qualification of healthcare providers
- Attribute levels such as longer distance/ shorter distance and qualified providers/non-qualified providers
- Factors influencing these preferences, such as age, income, education, and perceived severity of illness
- Studies that do not report specific preference attributes or attribute levels related to PHC providers will be excluded
- Studies that consider shared decision-making for preference (include providers in the process) will be excluded
- Studies that assessed the preference of alternative treatment options in PHC settings such as treatment A versus treatment B will be excluded

**Types of studies**

- Studies utilizing stated preference methods such as discrete choice experiments (DCE), conjoint analysis
- Cross-sectional studies applying either quantitative or qualitative approaches to explore preference
- Mixed-method studies combining qualitative and quantitative approaches to explore preference
- Studies published in English in any year
- Study protocols, newspaper articles, letters, editorials, personal communications, and commentaries, conference papers, systematic reviews, and scoping reviews will be excluded

**Context or settings**

- Studies conducted in LMICs
- Studies focusing on urban populations, or those comparing preferences for choosing healthcare provider between urban and rural populations
- Studies conducted focusing on PHC preference either in community or at healthcare facility settings
- Studies focusing on the preference for higher-level healthcare / specialized care will be excluded
- Studies that exclusively focus on rural populations without a comparative urban component will be excluded, as the primary interest is in the urban context

**Appendix 4. Detailed characteristics of included studies**

| **Sl** | **Author** | **Country** | **Study design** | **Study population and condition** | **Sample size** | **Recruitment settings and type of visits** | **Number of attributes** | **List of attributes/ factors examined or identified** | **Most Valued attributes** | **Factors affecting preference heterogeneity** |
| --- | --- | --- | --- | --- | --- | --- | --- | --- | --- | --- |
| 1 | Leslie, HH et al. (2023)  DOI: 10.1001/jamanetworkopen.2023.2937 | India | SP | Adult, Hypertension | 1085 | Community, Outpatient | 5 | - Staff attitude - Total wait time - Clinician type - Quality of clinical assessment - Availability of free medication | Availability of free medication | N/A |
| 2 | Wang, H et al. (2022)  DOI: 10.2147/PPA.S371188 | China | SP | Adult, Chronic disease | 248 | Community, Outpatient | 7 | - Follow-up frequency - Medicine accessibility - Family doctor competency - Health management - Referral convenience - Appointment flexibility - Shared decision making | Medicine accessibility | N/A |
| 3 | Li, Xia et al. (2021)  DOI: 10.3389/fpubh.2021.665282 | China | RP | Adult, General care | 728 | Community, Both (outpatient and inpatient) | 10 | - Doctor' s techniques/skills - Medical equipment - Medical costs - Service attitude - Range of medicines - Environmental conditions - Waiting time - Convenience of procedures - Services rationality - Convenient distance | Convenient distance | Gender, age, education, employment status, marital status, annual family income, insurance, location, severity of illness, type of illness, type of care |
| 4 | Khoo, EJ et al. (2021) DOI: 10.1080/13575279.2021.1920368 | Malaysia | RP | Parents of pediatric patient, General pediatric condition | 387 | Facility, Outpatient | 5 | - Consultation time - Facilities and services - Fees and charges - Social media engagement - Doctors' appearance | Consultation time | Ethnicity, income, age, gender, education, insurance |
| 5 | Liu, Y et al. (2020)  DOI: 10.1093/heapol/czz159 | China | SP | Adult, General care | 532 | Community, Outpatient | 7 | - Time taken for a visit - Out-of-pocket expense for visit - Medical professionals’ skill - Personal connection in the hospital - Medical equipment condition - Travel time from home to hospital - Hospital size | Time taken for a visit | Severity of illness |
| 6 | Khatami, F et al. (2020)  DOI: 10.1186/s12875-020-01181-2 | Iran | RP | Adult, General care | 400 | Facility, Not specified | 26 | - Being a specialist - Careful examination with receiving a detailed medical history - Sufficient time assigned to visit - Physicians’ intelligible expression - Providing services other than visit like injection - Rational prescription of drugs and tests - Health and illness status - Regular follow-up of patients’ illness - High trust in the doctor - Attention to other aspects of health - Keeping the patient’s medical secrets - Good morals and behavior - Proximity to the doctor’s office - Kindness and empathy with the patient - The receptionist’s or secretary’s behavior - Well-equipped and stylish doctor’s office - Waiting time to the doctor’s visit - Satisfaction of other patients Doctor’s reputation - Doctor’s reputation - Doctor’s age and experience - Recommendation of health care workers - More time accessibility to the doctor - Suggestion by friends and acquaintances - Physician’s appearance - Being same gender - Being a fellow-citizen | Type of provider | Gender |
| 7 | Jiang, MZ (2020) DOI: 10.1186/s12913-020-05134-4 | China | SP | Adult, Primary healthcare | 2019 | Facility, Outpatient | 4 | - Care provider - Mode of service - cost - Travel time | Travel time | Severity, Insurance, education, age, gender |
| 8 | Wang, X et al. (2020) DOI: 10.3390/ijerph17010117 | China | SP | Adult, Type 2 diabetes | 307 | Facility, Outpatient | 8 | - Attentiveness Of care provider - Attention to personal situation - Multidisciplinary care - Experience Of care provider - Accurate health information - Friendliness and helpfulness of staff - Travel time to care provider - Out Of pocket costs | Travel time to care provider | N/A |
| 9 | Zhu, J et al. (2019) DOI: 10.1002/hpm.2841 | China | SP | Adult, Common illness and Diabetes | 704 | Community, Outpatient | 5 | - Increment in Medicare reimbursement - Waiting time reduction - Prior expert treatment - Relationship with GP - Medicine and equipment in community health sector | Medicine and equipment in community health sector | Education, Income, number of visits, gender, age |
| 10 | Černauskas et al. (2018) doi: 10.1186/s12913-018-3264-x | India | SP | Adult, Not specified | 93 | Community, Outpatient | 5 | - Provider type and cost - Distance to the facility - Attitude of doctor and staff towards the patient - Appropriateness of care - Familiarity with the doctor | Appropriateness of care | Education, age, gender, and marital status |
| 11 | Wu, D et al. (2017) doi: 10.1093/fampra/cmw133 | China. | RP | Adult, General care | 1248 | Community, Outpatient | 21 | - Lower out-of-pocket payments - Close proximity to home or workplace - Short waiting time - Better doctor-patient communication - Practitioners’ friendly or sincere attitudes - Trust in PCPs’ competence - Perceived scale of the facility - Reliable medication - Expectation of diagnostic tests - Expectation of recovery - Freedom of choice - Level of sophistication of medical equipment - Availability of medication - Severity of illness - Reputation of the facility - Average education Of doctors - On-site waiting time - Distance - Family or friend's recommendation - Personal connections with doctors - Opportunity costs due to seeing a doctor | Level of sophistication of medical equipment | Perceived severity of the child’s illness and education |
| 12 | Boachie, MK (2015) doi: 10.15171/IJHPM.2015.191 | Ghana | RP | Adult, Hypertension | 496 | Community, Outpatient | 8 | - Availability of essential drugs - Distance or proximity - Recommendations from friends, co-workers - Availability of doctors - Shorter waiting time - Provider reputation - Clean environment - Presence of additional charges aside insurance | Availability of essential drugs | Gender, age, education, income, residence |
| 13 | Galal, SB et al. (2014) DOI: 10.1016/j.jegh.2013.12.002 | Egypt | RP | children under 18 years, General care | 1349 | Community, Not specified | 6 | - Good quality service - Near their home - Know health care providers - Free services - Suitable working hours - Others | Good quality service (urban)  Near their home (rural) | N/A |
| 14 | C. Tang et al. (2013) DOI: 10.1007/s10900-013-9691-z | China | RP | Adult, General care | 849 | Facility, Outpatient | 8 | - Convenience of seeking healthcare - Reasonable medical charges - Medical quality - Medical equipment - Variety of drugs - Attitude of the doctor - Loyalty/familiarity to the doctor - Recommended by others | Convenience of seeking healthcare | Location, Education, insurance type, area |
| 15 | D. Qian et al. (2010) DOI: 10.1016/j.healthpol.2010.06.014 | China | RP | All age groups, acute upper respiratory tract infections | 46510 | Community, Outpatient | 3 | - Distance - Price - Quality of care | Distance | Age, gender, marital status, education, occupation, consumption, city size, severity of illness, insurance status |
| 16 | Lungu, EA et al. (2012) DOI: 10.1371/journal.pone.0189940 | Malawi | SP | Under-5, childhood illnesses | 301 | Community, Outpatient | 6 | - Distance to health facility - Availability of medicines and supplies - Waiting time - Attitude of health worker - Thoroughness of physical examination of the child - Cost of healthcare | Availability of medicines and supplies | N/A |
| 17 | Jacobsen, KH et al,.(2012) DOI: 10.1016/j.inhe.2012.09.004 | Sierra Leone | RP | Women aged 16 years and above, General care | 1091 | Community, Not specified | 3 | - Reputation - Cost - Location | Reputation | N/A |
| 18 | Amaghionyeodiwe, LA et al. (2007) DOI: 10.1007/s10729-007-9038-3 | Nigeria | RP | Adult, General care | 7920 | Community, Both (outpatient and inpatient) | 5 | - Better treatment - Cost (money price) of treatment - Accessibility/proximity (distance) - Income - Delay in treatment (time factor) | Accessibility/proximity (distance) | Type of household head, education, age, nature of illness, income |
| 19 | Chiwire, P et al. (2022) DOI: 10.3390/ijerph19010590 | South Africa | SP | Adult, General care | 463 | Facility, Outpatient | 6 | - Distance to health facility - Confidentiality during treatment - Waiting time for the first visit to the facility (without an appointment) - Waiting time with an appointment - Treatment offered by doctor or nurse - Availability of required medication | Availability of required medication | Gender and type of hospital |
| 20 | Sun, X et al. (2019) DOI: 10.1371/journal.pone.0211984 | China | RP | Adult, General care | 915 | Community, Not specified | 7 | - Convenience of care - Reasonable charges - Good quality of care - Trust in doctors - Medical insurance designated facility - Good patient-doctor communication - Prior experience with the doctors | Convenience of care | Age, marital status, employment status, education, insurance, income, self-reported health status |
| 21 | Abodunrin, O. L. et al. (2010) DOI: 10.4314/ijhr.v3i2.70271 | Nigeria | RP | Adult, General care | 366 | Community, Not specified | 11 | - Cheap Service - Convenience/Proximity - Qualified Personnel - Quick Service - Privacy - Good Attitude of Staff - Equipment and lab Service - Drug Availability - Family Hospital - Other reasons - No other Choice | Quick Service | Sex, marital status, education, occupation, area |
| 22 | Jiang, S et al. (2020) DOI: 10.1016/j.chieco.2020.101538 | China | RP | Adult, General care | 202 | Both community and facility, Not specified | 7 | - Cost - Type of physician - Test/examinations availability - Variety of medications - Travel time to healthcare facility - Total visit time in healthcare facility - Friendliness of doctors | a) Tests/examinations availability (community resident:)  b) Type of physician (hospital patients)  c) Costs (clinic patients) | N/A |
| 23 | Lv Y., Qin j., Feng X., Li S., Tang C., & Wang H. (2023) doi:10.1136/bmjopen-2023-072496 | China | SP | Adult, Diabetes mellitus | 829 | Community, Not specified | 5 | - Medical service capacity - Out-of-pocket medical costs per month - Travel time - Attitude of medical staff   Availability of diabetes drugs | Medical service capacity | Region, gender, age, education, Employment status, Family income, duration of illness, complication status, family history, multimorbidity |
| 24 | Porter JD and Bresick G. (2017) doi:10.4102/phcfm.v9i1.1222 | South Africa | RP | Adult, General care | 164 | Community, Outpatient | 15 | - Staff treated with respect - Friendly staff - Take time to listen - Easier to see a doctor - Provide correct treatment - Recommended by others - Trust and confidentiality - Christian clinic - Treat children well - Seen by the same doctor - Cleanliness of the facility - Native language - Proximity of the clinic - Affordable services | Staff treat with respect (behavior of provider) | N/A |
| 25 | Nguyen HTT et al. (2022)  doi: 10.1002/hpm.3599 | Vietnam | SP | Adult, General care | 822 | Community, Not specified | 5 | - Visit duration - Travel time - Health insurance - Personal connection - Doctor's experience | Visit duration | N/A |
| 26 | Jia E et al. (2020)  doi:10.3390/ijerph17113988 | China | SP | Adult, Non-communicable disease | 196 | Community, Not specified | 5 | - Type of service - Treatment measures - Cost (CNY) - Travel time - Care provider | Tratment measures (major conditions)  Travel time (minor conditions) | Marital status, family per capita monthly income, region, gender, and employment (major conditions); Region, gender, education (minor conditions) |
| 27 | Peng Y et al. (2020)  doi: 10.2147/PPA.S265093 | China | SP | Adult, Chronic disease | 372 | Community, Outpatient | 5 | - Types of service - Treatment options - OOP cost per visit (CNY) - Distance to practice - Seniority of medical practitioner | Distance to practice | Gender, age, education, income, the region of residence, and status of chronic conditions |
| 28 | Li X et al. (2021)  doi: 10.1186/s13690-021-00728-9 | China | SP | Adult, Chronic disease | 680 | Primary care facility, Outpatient | 5 | - Types of service - Treatment options - OOP cost per visit (CNY) - Distance to practice - Seniority of medical practitioner | Treatment options | N/A |

**Appendix 5. Methodological quality ratings of included studies, based on ISPOR Task Force for Conjoint Analysis checklist**

|  |  | **Number of selected studies** | | | | | | | | | | | | | | |
| --- | --- | --- | --- | --- | --- | --- | --- | --- | --- | --- | --- | --- | --- | --- | --- | --- |
| **Item** | **Description** | **8** | **5** | **22** | **19** | **9** | **2** | **1** | **10** | **7** | **16** | **23** | **25** | **26** | **27** | **28** |
| A | Was a well-defined research question stated and is DCE an appropriate method for answering it? | Y | Y | Y | Y | Y | Y | Y | Y | Y | Y | Y | Y | Y | Y | Y |
| A(i) | Were a well-defined research question and a testable hypothesis articulated? | Y | Y | Y | Y | Y | Y | Y | Y | Y | Y | Y | Y | Y | Y | Y |
| A(ii) | Was the study perspective described, and was the study placed in a particular decision-making or policy context? | Y | Y | Y | Y | Y | Y | Y | Y | Y | Y | Y | Y | Y | Y | Y |
| A(iii) | What is the rationale for using conjoined analysis to answer the research question? | Y | Y | Y | Y | Y | Y | Y | Y | Y | Y | Y | Y | Y | Y | Y |
| B | Was the choice of attributes and levels supported by evidence? | Y | Y | P | Y | Y | Y | Y | Y | Y | Y | Y | Y | Y | Y | Y |
| B(iv) | Was attribute identification supported by evidence (literature reviews, focus groups or other scientific methods)? | Y | Y | Y | Y | Y | Y | Y | Y | Y | Y | Y | Y | Y | Y | Y |
| B(v) | Was attribute selection justified and consistent with theory? | Y | Y | Y | Y | Y | Y | Y | Y | Y | Y | Y | Y | Y | Y | Y |
| B(vi) | Was level selection for each attribute justified by the evidence and consistent with the study perspective and hypothesis? | P | Y | Y | Y | Y | Y | Y | Y | Y | Y | Y | Y | Y | Y | Y |
| C | Was the construction of tasks appropriate? | P | Y | P | N | Y | P | P | P | P | Y | P | P | P | P | P |
| C(vii) | Was the number of attributes in each conjoint task justified (that is, full or partial profile)? | Y | Y | Y | N | Y | Y | Y | Y | Y | Y | Y | Y | Y | Y | Y |
| C(viii) | Was the number of profiles in each conjoint task justified? | Y | Y | Y | N | Y | Y | Y | Y | Y | Y | Y | Y | Y | Y | Y |
| C(ix) | Was (should) an opt-out or a status-quo alternative (be) included? | N | Y | N | N | Y | N | N | N | N | Y | N | N | N | N | N |
| D | Was the choice of experimental design justified and evaluated? | P | N | Y | N | Y | Y | N | P | P | N | Y | Y | Y | Y | Y |
| D(x) | Was the choice of experimental design justified? Were alternative experimental designs considered? | Y | N | Y | Y | Y | Y | N | Y | N | N | Y | Y | Y | Y | Y |
| D(xi) | Were the properties of the experimental design evaluated? | N | N | Y | N | Y | Y | N | N | N | N | Y | Y | Y | Y | Y |
| D(xii) | Was the number of conjoint tasks included in the data collection instrument appropriate? | Y | N | Y | Y | Y | Y | Y | Y | Y | Y | Y | Y | Y | Y | Y |
| E | Were preferences elicited appropriately, given the research question? | P | N | P | P | P | P | P | P | Y | P | Y | P | Y | Y | Y |
| E(xiii) | Was there sufficient motivation and explanation of conjoint tasks? | Y | N | Y | N | Y | Y | Y | Y | Y | Y | Y | Y | Y | Y | Y |
| E(xiv) | Was an appropriate elicitation format (that is, rating, ranking, or choice) used? Did (should) the elicitation format allow for indifference? | Y | P | Y | Y | N | Y | Y | Y | Y | Y | Y | Y | Y | Y | Y |
| E(xv) | In addition to preference elicitation, did the conjoint tasks include other qualifying questions (for example, strength of preference, confidence in response, and other methods)? | N | N | N | Y | Y | N | N | N | Y | P | Y | N | Y | Y | Y |
| F | Was the data collection instrument designed appropriately? | Y | P | P | P | Y | Y | P | Y | Y | P | Y | Y | Y | Y | Y |
| F(xvi) | Was appropriate respondent information collected (such as sociodemographic, attitudinal, health history or status, and treatment, experience)? | Y | Y | Y | Y | Y | Y | Y | Y | Y | Y | Y | Y | Y | Y | Y |
| F(xvii) | Were the attributes and levels defined, and was any contextual information provided? | P | N | Y | N | Y | Y | P | Y | Y | P | Y | Y | Y | Y | Y |
| F(xviii) | Was the level of burden of the data-collection instrument appropriate? Were respondents encouraged and motivated? | P | Y | N | N | Y | Y | N | Y | Y | P | Y | Y | Y | Y | Y |
| G | Was the data collection plan appropriate? | Y | P | Y | P | P | Y | Y | Y | Y | Y | Y | Y | Y | Y | Y |
| F(xix) | Was the sampling strategy justified (for example, sample size, stratification, and recruitment)? | Y | P | Y | P | Y | Y | Y | Y | P | Y | Y | Y | Y | Y | Y |
| F(xx) | Was the mode of administration justified and appropriate (for example, face-to-face, pen-and-paper, web-based)? | Y | Y | Y | N | Y | Y | Y | Y | Y | Y | Y | Y | Y | Y | Y |
| F(xxi) | Were ethical considerations addressed (for example, recruitment, information and/or consent, compensation)? | Y | Y | Y | Y | N | Y | Y | Y | Y | Y | Y | Y | Y | Y | Y |
| H | Were statistical analyses and model estimations appropriate? | P | Y | Y | P | P | Y | Y | Y | Y | P | Y | Y | Y | Y | Y |
| H(xxii) | Were respondent characteristics examined and tested? | Y | Y | Y | Y | Y | Y | Y | Y | Y | Y | Y | Y | Y | Y | Y |
| H(xxiii) | Was the quality of the responses examined (for example, rationality, validity, reliability)? | N | Y | Y | N | N | Y | Y | Y | Y | P | Y | Y | Y | Y | Y |
| H(xxiv) | Was model estimation conducted appropriately? Were issues of clustering and subgroups handled appropriately? | Y | Y | Y | Y | Y | P | Y | Y | Y | Y | Y | Y | Y | Y | Y |
| I | Were the results and conclusions valid? | Y | Y | Y | Y | Y | Y | Y | Y | Y | Y | Y | Y | Y | Y | Y |
| I(xxv) | Did study results reflect testable hypotheses and account for statistical uncertainty? | Y | Y | Y | Y | Y | Y | Y | Y | Y | Y | Y | Y | Y | Y | Y |
| I(xxvi) | Were study conclusions supported by the evidence and compared with existing findings in the literature? | Y | Y | Y | Y | Y | Y | Y | Y | P | Y | Y | Y | Y | Y | Y |
| I(xxvii) | Were study limitations and generalizability adequately discussed? | Y | Y | Y | Y | Y | Y | Y | Y | Y | P | Y | Y | Y | Y | Y |
| J | Was the study presentation clear, concise and complete? | Y | Y | Y | Y | Y | Y | Y | Y | Y | Y | Y | Y | Y | Y | Y |
| J(xxviii) | Was study importance and research context adequately motivated? | Y | Y | Y | Y | Y | Y | Y | Y | Y | Y | Y | Y | Y | Y | Y |
| J(xxix) | Were the study data-collection instrument and methods described? | Y | Y | Y | Y | Y | Y | Y | Y | P | Y | Y | Y | Y | Y | Y |
| J(xxx) | Were the study implications clearly stated and understandable to a wide audience? | Y | Y | Y | Y | Y | Y | Y | Y | Y | Y | Y | Y | Y | Y | Y |
| Total main items rated “Yes” | | 7 | 6 | 6 | 4 | 7 | 8 | 6 | 7 | 8 | 6 | 9 | 8 | 9 | 9 | 9 |
| % main items rated “Yes” | | 70 | 60 | 60 | 40 | 70 | 80 | 60 | 70 | 80 | 60 | 90 | 80 | 90 | 90 | 90 |
| Total main items rated “Partial” | | 3 | 2 | 4 | 4 | 3 | 2 | 3 | 3 | 2 | 3 | 1 | 2 | 1 | 1 | 1 |
| % main items rated “Partial” | | 30 | 20 | 40 | 40 | 30 | 20 | 30 | 30 | 20 | 30 | 10 | 80 | 10 | 10 | 10 |
| Total main items rated “No” | | 0 | 2 | 0 | 2 | 0 | 0 | 1 | 0 | 0 | 1 | 0 | 0 | 0 | 0 | 0 |
| % main items rated “No” | | 0 | 20 | 0 | 20 | 0 | 0 | 10 | 0 | 0 | 10 | 0 | 0 | 0 | 0 | 0 |

**Appendix 6: Methodological quality ratings of included studies, based on mixed method meta-analysis appraisal tool (MMAT)**

| **Criterion** | **Number of selected studies** | | | | | | | | | | | |
| --- | --- | --- | --- | --- | --- | --- | --- | --- | --- | --- | --- | --- |
|  | **6** | **21** | **20** | **18** | **17** | **12** | **4** | **13** | **14** | **15** | **11** | **24** |
| S1. Are there clear research questions? | Y | Y | Y | Y | Y | Y | Y | Y | Y | Y | Y | Y |
| S2. Do the collected data allow to address the research questions? | Y | Y | Y | Y | Y | Y | Y | Y | Y | Y | Y | Y |
| **Mixed methods** |  |  |  |  |  |  |  |  |  |  |  |  |
| 1.1. Is there an adequate rationale for using a mixed methods design to address the research question? | Y |  |  |  |  | Y |  |  |  |  | Y | Y |
| 1.2. Are the different components of the study effectively integrated to answer the research question? | Y |  |  |  |  | Y |  |  |  |  | Y | Y |
| 1.3. Are the outputs of the integration of qualitative and quantitative components adequately interprete1 | Y |  |  |  |  | Y |  |  |  |  | Y | Y |
| 1.4. Are divergences and inconsistencies between quantitative and qualitative results adequately addressed? | CT |  |  |  |  | N |  |  |  |  | N | Y |
| 1.5. Do the different components of the study adhere to the quality criteria of each tradition of the methods involved? | Y |  |  |  |  | Y |  |  |  |  | Y | Y |
| **Qualitative study** |  |  |  |  |  |  |  |  |  |  |  |  |
| 1.1. Is the qualitative approach appropriate to answer the research question? | Y |  |  |  |  | Y |  |  |  |  | Y | Y |
| 1.2. Are the qualitative data collection methods adequate to address the research question? | Y |  |  |  |  | Y |  |  |  |  | Y | Y |
| 1.3. Are the findings adequately derived from the data? | Y |  |  |  |  | Y |  |  |  |  | Y | Y |
| 1.4. Is the interpretation of results sufficiently substantiated by data? | Y |  |  |  |  | Y |  |  |  |  | Y | N |
| 1.5. Is there coherence between qualitative data sources, collection, analysis and interpretation? | Y |  |  |  |  | Y |  |  |  |  | Y | CT |
| **Quantitative descriptive study** |  |  |  |  |  |  |  |  |  |  |  |  |
| 1.1. Is the sampling strategy relevant to address the research question? | Y | Y | Y | Y | Y | Y | Y | Y | Y | Y | Y | Y |
| 1.2. Is the sample representative of the target population? | Y | Y | Y | Y | Y | Y | Y | Y | Y | Y | Y | Y |
| 1.3. Are the measurements appropriate? | Y | Y | Y | Y | Y | Y | Y | Y | Y | Y | Y | Y |
| 1.4. Is the risk of nonresponse bias low? | CT | Y | CT | Y | CT | CT | CT | N | Y | Y | Y | N |
| 1.5. Is the statistical analysis appropriate to answer the research question? | Y | CT | Y | Y | Y | Y | Y | N | Y | Y | Y | Y |

| Y | Rated “Yes” |
| --- | --- |
| P | Rated “Partial” |
| N | Rated “No” |

**Appendix 7: Direction of commonly reported heterogenous factors**

| **No.** | **Study** | **Type of study** | **Illness severity / health status** | **Gender** | **Education** | **Income** | **Age** | **Summary of heterogeneity direction** |
| --- | --- | --- | --- | --- | --- | --- | --- | --- |
| 3 | (Li et al. 2021) | Non-DCE | Severe illness ↓ PHC provider | – | Higher ↓ PHC provider | Higher ↓ PHC provider | Older ↑ PHC provider | Older, lower groups more likely to choose PHC provider |
| 4 | (Khoo et al. 2021) | Non-DCE | – | Men ↑ doctors’ appearance | Tertiary ↑ shared decision-making | Lower ↑ facility attributes | – | Gender, education and income influence preference attributes |
| 5 | (Liu et al. 2020) | DCE | Minor ↑ access; Severe ↑ quality | – | – | – | – | Severity shifts trade-offs from convenience to quality |
| 6 | (Khatami et al. 2020) | DCE | – | Female ↑ communication skills of providers | Higher ↑ technical quality | – | – | Gender and education differentiate interpersonal vs quality priorities |
| 7 | (Jiang et al. 2020) | DCE | Poor health ↑ higher-level care | – | Higher ↑ technology | Higher ↑ cost tolerance | Older ↑ higher-level care | Health status and SES favour higher-level facilities |
| 9 | (Zhu et al. 2019) | DCE | Chronic ↑ continuity of care | Female ↑ known provider | Higher ↑ specialist care | Lower ↑ reimbursement | Older ↑ reimbursement | Structured heterogeneity across SES and health status |
| 10 | (Černauskas et al. 2018) | DCE | – | Female ↑ familiarity | Higher ↑ appropriateness | Higher ↑ modern providers | Older ↑ modern care | Education/income shift preferences to technical care |
| 11 | (Wu et al. 2017) | Non-DCE | Higher severity ↑ hospital | – | – | – | Children ↑ hospital | Severity drives hospital choice |
| 12 | (Boachie 2016) | Non-DCE | – | Male ↓ private provider | – | Higher ↓ public provider | – | Factors influence choice of public and private provider |
| 14 | (Tang et al. 2013) | Non-DCE | – | – | Higher ↓ PHC provider | – | Older ↑ PHC provider | Higher education negatively and older age positively influence |
| 15 | (Qian et al. 2010) | Non-DCE | Severe ↑ hospital | – | – | Higher ↑ hospital | Children ↑ hospital | Severity and income dominate provider choice |
| 18 | (Amaghionyeodiwe 2008) | Non-DCE | – | – | – | Higher ↑ modern care | Older ↑ public and private | Cost and distance constrain modern care |
| 19 | (Chiwire et al. 2022) | DCE | – | Female ↑ confidentiality, short waiting time | – | – | – | Gender and age shape behavioural/time preferences |
| 20 | (Sun et al. 2019) | Non-DCE | Poor health ↓ PHC provider | – | Higher ↓ PHC provider | Higher ↓ PHC provider | – | Accessibility and health status drive PCF use |
| 21 | (Abodunrin et al. 2010) | Non-DCE | – | Female ↑ PHC provider | Higher ↓ PHC provider | – | – | SES and residence shape facility choice |
| 23 | (Lv et al. 2023) | DCE | Longer duration of illness ↑ quality | Female ↑ attitude and travel time | Higher ↓ Travel time | Higher ↑ service capacity | Higher ↑ service capacity | Disease duration and income modify trade-offs |
| 26 | (Jia et al. 2020) | DCE | Severe ↑ senior/specialised | – | – | Higher ↑ quality | Older ↑ senior care | Severity dominates provider preferences |
| 27 | (Peng et al. 2020) | DCE | Chronic status ↑ Traditional Provider | Gender varies by class | Education ↑ Modern medicine | Higher ↑ Modern medicine | Older ↑ Modern medicine | Latent classes reflect structured heterogeneity |
